# Mitochondrial respiration during normothermic liver machine perfusion predicts clinical outcome

**DOI:** 10.1016/j.ebiom.2022.104311

**Published:** 2022-10-29

**Authors:** Andras T. Meszaros, Julia Hofmann, Madita L. Buch, Benno Cardini, Theresia Dunzendorfer-Matt, Florian Nardin, Michael J. Blumer, Margot Fodor, Martin Hermann, Bettina Zelger, Giorgi Otarashvili, Melanie Schartner, Annemarie Weissenbacher, Rupert Oberhuber, Thomas Resch, Jakob Troppmair, Dietmar Öfner, Heinz Zoller, Herbert Tilg, Erich Gnaiger, Theresa Hautz, Stefan Schneeberger

**Affiliations:** a Department of Visceral, Transplant and Thoracic Surgery, organLife™ Laboratory and Daniel Swarovski Research Laboratory, Center of Operative Medicine, Medical University of Innsbruck, Innsbruck, Austria; b Institute of Biological Chemistry, Biocenter, Medical University of Innsbruck, Innsbruck, Austria; c Department of Anatomy, Histology and Embryology, Division of Clinical and Functional Anatomy, Medical University of Innsbruck, Innsbruck, Austria; d Institute of Pathology, Neuropathology and Molecular Pathology, Medical University of Innsbruck, Innsbruck, Austria; e Department of Internal Medicine I, Medical University of Innsbruck, Innsbruck, Austria; f Oroboros Instruments, Innsbruck, Austria

**Keywords:** Liver, Transplantation, Normothermic machine perfusion, Mitochondria, High-resolution respirometry, ADP, Adenosine diphosphate, ALT, Alanine aminotransferase, AMP, Adenosine monophosphate, AST, Aspartate aminotransferase, ATP, Adenosine triphosphate, AUC, Area under the curve, BMI, Body mass index, CCasp3, Cleaved caspase 3, DBD, Donation after brain death, DCD, Donation after cardiocirculatory death, DRI, Donor risk index, EAD, Early allograft dysfunction, ECD, Extended criteria donor, ET, Electron transfer, FAO, Fatty acid oxidation, FCR, Flux control ratio, FMN, Flavin mononucleotide, GLDH, Glutamate dehydrogenase, H&E, Haematoxylin and eosin, HOPE, Hypothermic Oxygenated machine Perfusion, HRR, High-resolution respirometry, IHC, Immunohistochemistry, IL-6, Interleukin 6, IRI, Ischemia-reperfusion injury, LDH, Lactate dehydrogenase, L-GrAFT, Liver Graft Assessment Following Transplantation, LT, Liver transplantation, MEAF, Model for Early Allograft Function, MELD, Model of End Stage Liver Disease, MP, Machine perfusion, mtIM, Mitochondrial inner membrane, mtOM, Mitochondrial outer membrane, NAFLD, Non-alcoholic fatty liver disease, NMP, Normothermic machine perfusion, OXPHOS, Oxidative phosphorylation, PI, Propidium iodidide, RTCM, Real-time confocal microscopy, SCS, Static cold storage, SD, Standard deviation, SUIT, Substrate-Uncoupler-Inhibitor Titration, TEM, Transmission electron microscopy, TLR4, Toll-like receptor 4, TNFα, Tumor necrosis factor alpha, WGA, Wheat germ agglutinin

## Abstract

**Background:**

Reliable biomarkers for organ quality assessment during normothermic machine perfusion (NMP) are desired. ATP (adenosine triphosphate) production by oxidative phosphorylation plays a crucial role in the bioenergetic homeostasis of the liver. Thus, detailed analysis of the aerobic mitochondrial performance may serve as predictive tool towards the outcome after liver transplantation.

**Methods:**

In a prospective clinical trial, 50 livers were subjected to NMP (OrganOx Metra) for up to 24 h. Biopsy and perfusate samples were collected at the end of cold storage, at 1 h, 6 h, end of NMP, and 1 h after reperfusion. Mitochondrial function and integrity were characterized by high-resolution respirometry (HRR), AMP, ADP, ATP and glutamate dehydrogenase analysis and correlated with the clinical outcome (L-GrAFT score). Real-time confocal microscopy was performed to assess tissue viability. Structural damage was investigated by histology, immunohistochemistry and transmission electron microscopy.

**Findings:**

A considerable variability in tissue viability and mitochondrial respiration between individual livers at the end of cold storage was observed. During NMP, mitochondrial respiration with succinate and tissue viability remained stable. In the multivariate analysis of the 35 transplanted livers (15 were discarded), area under the curve (AUC) of LEAK respiration, cytochrome *c* control efficiency (mitochondrial outer membrane damage), and efficacy of the mitochondrial ATP production during the first 6 h of NMP correlated with L-GrAFT.

**Interpretations:**

Bioenergetic competence during NMP plays a pivotal role in addition to tissue injury markers. The AUC for markers of outer mitochondrial membrane damage, ATP synthesis efficiency and dissipative respiration (LEAK) predict the clinical outcome upon liver transplantation.

**Funding:**

This study was funded by a Grant from the In Memoriam Dr. Gabriel Salzner Stiftung awarded to SS and the 10.13039/501100009968Tiroler Wissenschaftsfond granted to TH.

**Research in context**

**Evidence before this study**

Normothermic machine perfusion (NMP) enables for viability testing of the donor organ prior to transplantation. Intact mitochondrial respiration is crucial for adenosine triphosphate (ATP) production in the liver during NMP und after transplantation. Recently, the mitochondrial injury marker flavin mononucleotide (FMN) assessed during oxygenated cold perfusion, has been found to predict organ function. However, little is known about bioenergetic function and the predictive capacity of mitochondrial respiration during NMP. Herein, we characterized the mitochondrial respiratory capacity of the liver during NMP and assessed its predictive value towards the outcome after transplantation.

**Added value of this study**

Live cell confocal imaging demonstrated that tissue viability does not deteriorate during NMP. However, we observed a considerable variability of the mitochondrial respiration in human livers after cold ischemia by high-resolution respirometry. During NMP, the capacity of mitochondrial oxidative phosphorylation using the Complex II substrate succinate remains stable while fatty acid oxidation is decreasing. Bioenergetic function remains stable overall with preserved coupling of the respiration to ATP production and the mitochondrial outer membrane stays intact. In line with this, the energy charge increases after start of NMP. Nevertheless, some grafts exhibit declining coupling and increasing mitochondrial outer membrane damage during NMP. This was also visualized by transmission electron microscopy. Finally, we prove that monitoring the dissipative component of mitochondrial respiration, efficiency of ATP production, and mitochondrial outer membrane damage during the first 6 h of NMP predicts the clinical outcome of the transplantation.

**Implications of all the available evidence**

We characterized the substrate and coupling control of mitochondrial respiration in the liver during clinical NMP with high accuracy. Importantly, we could demonstrate the predictive capacity of mitochondrial respiratory function towards clinical outcome. Hence mitochondrial respiration may serve as a biomarker, which helps in the selection of organs suitable for liver transplantation.

## Introduction

The liver plays a central role in the metabolism of carbohydrates, lipids and proteins. Aerobic respiration is essential to cover the high energy demand. Thus, hepatocytes are rich in mitochondria^1^ and intact mitochondria are crucial for liver function. As illustrated in both experimental and clinical studies, liver pathologies such as non-alcoholic fatty liver disease (NAFLD) and ischemia-reperfusion injury (IRI) are characterized by impaired mitochondrial function.^2^^,^^3^ Over the last years, the extraordinary relevance of mitochondrial function in liver preservation and transplantation of organs has been recognised^4^ and a better understanding of the impact of mitochondrial damage on allograft dysfunction in response to ischemia-reperfusion has emerged.^5^ Due to the persistent shortage of organs for transplantation, the acceptance of extended criteria donor (ECD) livers has increased. However, ECD grafts are especially prone to pre-existing and secondary mitochondrial injuries.^6^^,^^7^ Most studies addressing mitochondrial function during machine perfusion (MP) in humans report only indirect measurements, such as damage-associated products in the perfusate and activities of isolated mitochondrial respiratory complexes.^8^^,^^9^ It has been demonstrated that mitochondrial respiration, mitochondrial membrane potential and intracellular adenosine triphosphate (ATP) can determine the liver quality prior of transplantation.^10^ However, data from direct assessment of mitochondrial function during MP in humans are scarce.

MP is an emerging preservation strategy for livers and other organs. The ability to assess organ function *ex vivo* is a key feature of normothermic machine perfusion (NMP) and may help to deem more organs as suitable for transplantation. While the advantage of MP is substantiated by an increasing number of studies, there is a need to find objective prognostic parameters for optimal organ selection.

Early attempts to identify biomarkers for organ viability and function mainly focused on the biochemical analysis of the perfusate.^11^, ^12^, ^13^ However, mitochondrial quality assessment in tissue biopsies has been recognized as an option in search of a sensitive parameter to predict organ function.^4^ There is expanding evidence in support of a potential correlation between mitochondrial injury and clinical outcome. Recently, Hypothermic Oxygenated machine Perfusion (HOPE) has been demonstrated to preserve or improve cellular bioenergetics.^9^ In a controlled multicenter trial, HOPE effectively reduced non-anastomotic biliary complications.^14^ Furthermore, a predictive value for the mitochondrial marker flavin mononucleotide (FMN) in the perfusate during MP towards the outcome has been described.^15^ However, the analysis of such metabolic products may indicate mitochondrial damage, but does not allow for evaluation of the actual bioenergetic function. While HOPE seems to result in bioenergetic reconditioning and FMN may serve for monitoring, the assessment of the quality and dynamics of bioenergetic function during NMP require a thorough analysis of mitochondrial respiration.

To this effect, we employed high-resolution respirometry (HRR) to characterize mitochondrial performance in small wedge biopsies after cold storage and during NMP. The method allows for real-time assessment of mitochondrial respiration, efficacy and damage in different metabolic pathways.^16^ We analysed mitochondrial parameters as biomarkers to predict the clinical outcome after transplantation. Herein we show a detailed characterization of the respiratory substrate- and coupling-control of mitochondrial respiration during human liver NMP. Collectively, our data suggest that mitochondrial respiration assessment during the early phase of liver NMP (up to 6 h) describes the bioenergetic function and predicts graft function after transplantation.

## Methods

### Study design and ethics

**Fig. 1:**
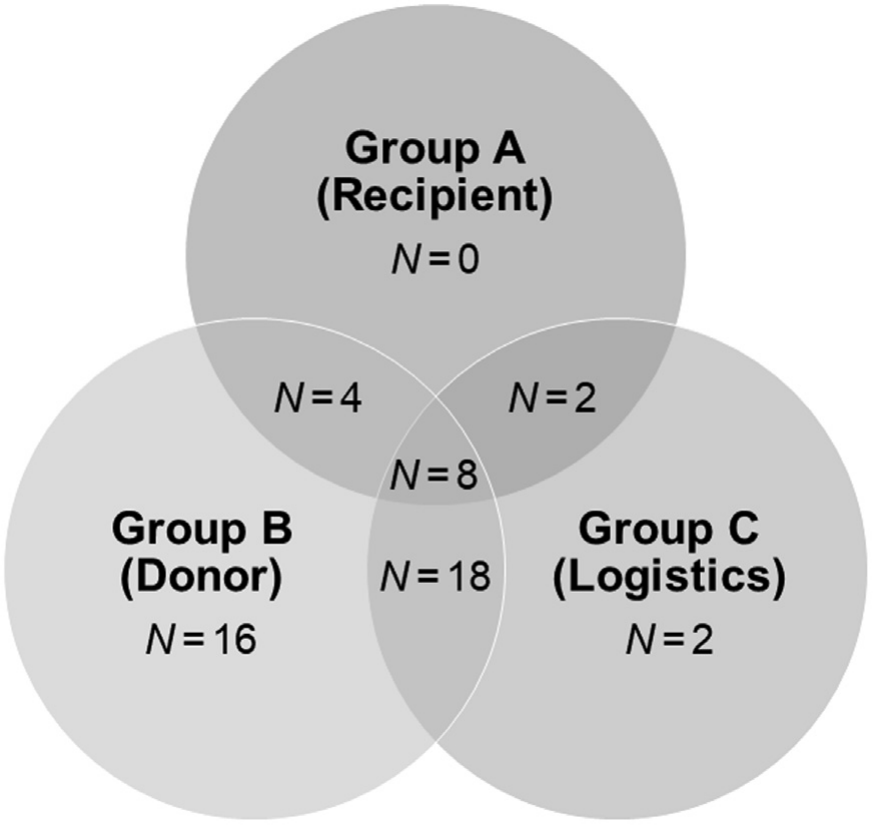
**Graphical representation of factors for decision to opt for normothermic machine perfusion.** A, patient factors; B, donor factors; C, logistic factors. See also Table 1.

50 patients were enrolled in a prospective, open-label, observational study between April 2019 and November 2020. The study was approved by the local ethics committee (Ethical committee of the Medical University of Innsbruck, EK Nr. 1175/2018) and all patients signed an informed consent form. Liver allografts were subjected to NMP prior to LT for one or a combination of the following indications: (1) suspected suboptimal organ quality (2) complex recipient, (3) logistics (details Table 1 and Fig. 1). For NMP, the protocol reported by Cardini et al. was followed.^17^ NMP times of up to 24 h (Metra®, OrganOx Limited, Oxford, United Kingdom) were allowed. Wedge biopsies were taken at the end of static cold storage (pre), 1 h, 6–9 h, 9–15 h, 15–24 h after the start of NMP and 1 h after reperfusion (post). Samples were immediately placed in ice-cold HTK (Custodiol®, Dr. Franz Köhler Chemie GmbH, Bensheim, Germany) and stored on ice until further processing. Perfusate samples were collected at 15 min, 1 h, 6 h, 12 h and 20 h after start of NMP. The last sample was taken at the end of NMP. Perfusate samples were analysed for aspartate aminotransferase (AST), alanine aminotransferase (ALT), lactate dehydrogenase (LDH), Interleukin 6 (IL-6), tumor necrosis factor alpha (TNFα) (all Roche Diagnostics GmbH, Mannheim, Germany), lactate (Drott Medizintechnik GmbH, Wiener Neudorf, Austria), and glutamate dehydrogenase (GLDH, see below).^11^ The decision for transplantation after NMP was based on the criteria previously described by our group^17^: (1) prompt decline to lactate levels ≤2.5 mmol L^−1^, (2) maintenance of physiological pH levels (7.30–7.45) without repeated sodium bicarbonate addition, (3) no high AST (>20,000 U L^−1^), ALT (>20,000 U L^−1^) or LDH (>20,000 U L^−1^) levels. Further to these criteria, IL-6 levels were recorded and excessively high levels were signal urging for caution.

**Table 1:** Factors for decision to opt for normothermic machine perfusion.

Indication for NMP	Total NMP cases	Result in transplant	Result in discard
A	0	0	0
B	16	9	7
C	2	2	0
A + B	4	4	0
A + C	2	2	0
B + C	18	10	8
A + B + C	8	8	0
**Total**	**50**	**35**	**15**

A, patient factors; B, donor factors; C, logistic factors.

In order to assess early allograft function, the risk scores Liver Graft Assessment Following Transplantation (L-GrAFT),^18^ Model for Early Allograft Function (MEAF)^19^ and early allograft dysfunction (EAD) according to Olthoff et al.^20^ were calculated.

### Mitochondrial respiration

HRR (O2k, Oroboros Instruments, Innsbruck, Austria) was employed to assess mitochondrial respiration. All measurements were carried out in 2 mL O2k chambers at 37 °C under constant stirring at 750 rpm.^16^ Data were acquired at a recording interval of 2 s and analysed with DatLab 7 software (Datlab 7.4, Oroboros Instruments, Innsbruck, Austria). Air-calibration was performed with MiR05 mitochondrial respiration medium (MiR05-Kit, Oroboros Instruments, Innsbruck, Austria, consisting of 0.5 mM EGTA, 3 mM MgCl_2_ 6 H_2_O, 60 mM lactobionic acid, 20 mM taurine, 10 mM KH_2_PO_4_, 20 mM HEPES, 110 mM d-sucrose, and addition of 1 g L^−1^ essentially fatty acid free bovine serum albumin) before each experiment. 20 mg liver tissue was dissected on a cooled plate at 4 °C, weighted and homogenized in 4 °C MiR05 using a PBI-Shredder O2k-Set (Oroboros Instruments, Innsbruck, Austria). 2 mL tissue homogenate with a final wet-mass of 1 mg mL^−1^ was immediately added into each of the O2k chambers. Chemicals for the pre-defined Substrate-Uncoupler-Inhibitor Titration (SUIT) protocols were titrated using glass microsyringes (Oroboros Instruments, Innsbruck, Austria). The protocols SUIT01 and SUIT02 are shown in the Supplementary Tables 1 and 2. Each titration step was carried out as soon respiration reached a steady state. Measurements were performed in duplicates.

Respiration rates were expressed as O_2_ flux per wet tissue mass [pmol O_2_ s^−1^ mg^−1^]. Respiratory capacities were normalized to an internal reference rate for each measurement to determine flux control ratios (FCR) for evaluation of SUIT01. For SUIT02, coupling states LEAK, OXPHOS, OXPHOS(c) and ET were evaluated.^21^ The following control efficiencies were calculated for the succinate pathway: *P-L* control efficiency (1-*L*/*P*), to evaluate the efficiency of ATP production; cytochrome *c* control efficiency (1-*P/P*_*c*_), to assess the damage to the mitochondrial outer membrane (mtOM); *E-P* control efficiency (1-*P/E*), to determine the control of the phosphorylation system over the electron transfer (ET) capacity.^21^

### Real-time confocal microscopy (RTCM)

Cell viability and tissue integrity were assessed using RTCM and evaluated according to a previously described scheme.^22^ Briefly, biopsies were incubated with SYTO®16 (green, staining of living cells, Molecular Probes; final concentration 5 μM), propidium iodide (PI; red, staining of dead cells, Molecular Probes; final concentration 500 nM) and wheat germ agglutinin (WGA; blue, staining of cell morphology, Molecular Probes, Eugene, OR, USA; final concentration 10 mM) for 15 min at room temperature. Images were acquired with a spinning disk confocal system (PerkinElmer, Wellesley, MA) mounted on an Olympus IX-70 inverse microscope (Olympus, Nagano, Japan) and collected using the ULTRA VIEW LCI software 5.4 (PerkinElmer, Wellesley, MA).

Quantification was based on a semi-quantitative score (0-1-2): 0 = highly viable cells, 1 = number of viable and dead cells equals, 2 = highly non-viable cells.

### Histology and immunohistochemistry (IHC)

Tissue biopsies were fixed in 10% formalin. After paraffine embedding, 4 μM sections were generated and Haematoxylin & Eosin (H&E) staining was performed according to standard protocols. The assessment of necrosis, steatosis, fibrosis, inflammation, and vascular changes was based on a semi-quantitative modified Suzuki score.^23^

The following immunohistochemical stainings were applied using commercially available and validated antibodies: CD3 (mouse monoclonal anti-CD3, dilution 1:100, Cat# MA1-80469, RRID:AB_928106, ThermoFisher, Waltham, MA, USA), CD68 (mouse monoclonal anti-CD68, Cat# MA5-13324, RRID:AB_10987212, ThermoFisher, Waltham, MA, USA),^24^ CD20 (mouse monoclonal anti-CD20, dilution 1:100, Cat# sc-70582, RRID:AB_1120279, Santa Cruz Biotechnology, Dallas, TX, USA), Toll-like receptor 4 (TLR4; mouse monoclonal anti-TLR4, dilution 1:200, Cat# ab22048, RRID:AB_446735, Abcam, Cambridge, UK), cleaved caspase 3 (CCasp3; polyclonal anti-CCasp3, dilution 1:400, Cat# 9661, RRID:AB_2341188, Cell Signaling Technology, Danvers, MA, USA).^25^ Scoring was based on the H-Score previously described elsewhere.^26^

### Ion pair chromatography

Snap frozen tissue biopsies (3–15 mg) were homogenized (T-10 ULTRA-TURAX, IKA, Staufen Germany) in 50 mM KH_2_PO_4_ (pH 6.2), supplemented with CH_3_CN (50% (v/v) and purified by centrifugation (17,000 × *g*, 4 °C). Supernatants of tissue lysates containing nucleotides were analysed in duplicates by ion pair chromatography using a reversed phase C18 HPLC column (LiChroCART 250 × 4.6) together with a mobile phase containing 100 mM KH_2_PO_4_ (pH 6.2), 35 mM tetrabutylammonium bromide, 7.5% (v/v) CH_3_CN under isocratic conditions. The signal was detected by UV absorbance at 260 nm. Standard solutions of adenosine monophosphate (AMP), adenosine diphosphate (ADP), and adenosine triphosphate (ATP) were injected in serial dilutions at eight different concentrations in triplicates and used to quantify the detected nucleotides. Ratio of ATP to ADP and energy charge defined as: (ATP + 0.5 ∗ ADP)/(ATP + ADP + AMP) were calculated as previously described.^27^

### Transmission electron microscopy (TEM)

Integrity of the mitochondrial membranes was verified by TEM. Liver biopsies (2 × 2 × 2 mm) were fixed in 2.5% glutaraldehyde and 2% paraformaldehyde buffered in sodium cacodylate (0.1 M, pH = 7.4) over night at 4 °C. Subsequently, they were rinsed in sodium cacodylate buffer and postfixed in 0.5% osmium tetroxide, 1% potassium hexacyanidoferrate in distilled water for 4 h at 4 °C. Again, samples were rinsed, dehydrated in graded ethanol series and embedded in EPON resin (# 45,359, Sigma–Aldrich, Austria). Ultrathin sections (90 nm) were cut on a Reichert Ultracut S microtome (Leica Microsystem, Wetzlar, Germany) with an ultra-diamond knife (Diatome, Biel, Switzerland), mounted on dioxan-formvar coated slot-grids (#G2500C, Christine Gröpl, Elektronenmikroskopie, Tulln, Austria) and stained with 1% uranyl acetate and lead citrate. The sections were examined with a Philips CM 120 transmission electron microscope at 80 kV (FEI, Eindhoven, Netherlands) equipped with a MORADA digital camera (Olympus SIS, Münster, Germany).

### Quantification of glutamate dehydrogenase (GLDH)

For analysis of GLDH activity in perfusate samples, a commercially available kit (ab102527, Abcam, Cambridge, UK) was used according to the manufacturer's instructions. Briefly, plasma samples were added to the reaction mix containing GLDH assay buffer, GLDH developer, and 2 M glutamate and incubated for 30 min. Absorbance was measured every 4 min for 2 h at 450 nm to determine the exponential phase of the kinetic curve. GLDH activity is expressed as U L^−1^.

### Statistical analysis

Shapiro–Wilk Test was applied to test for normality of distribution. Descriptive statistics are represented as median and interquartile ranges (IQR) or mean ± SD. To assess differences between groups Mann–Whitney U or Kruskal–Wallis with Dunn's multiple comparisons test for non-normally distributed continuous variables was used. Repeated measures ANOVA (RM ANOVA) with Tukey's multiple comparisons test was applied to assess differences within groups at different time points and two-way ANOVA with Šídák's multiple comparisons test was performed for two factors. Area under the curve (AUC) was calculated for mitochondrial parameters to include the time-component of machine perfusion using the R package ‘stats’. A linear relationship between the mitochondrial parameters and risk scores was assumed. Model variables with a predictive value towards the clinical outcome were chosen by akaike information criterion (AIC) in a stepwise algorithm using the R package ‘stats’. To confirm the models, type II ANOVA using the R package ‘car’ and multiple regression analysis using the R package ‘stats’ were applied. For all other statistical analyses and figures Graph Pad Prism 9 was used. *p*-values < 0.05 were considered as statistically significant.

### Role of funding source

The funding sources (In Memoriam Dr. Gabriel Salzner Stiftung and Tiroler Wissenschaftsfond) had no role in study design, data collection, data analysis, interpretation or writing of the manuscript.

## Results

### Normothermic machine perfusion, patient characteristics and outcome

A total of 50 human liver grafts undergoing NMP prior to LT were included. Of these, 35 livers were found suitable for transplantation according to the organ quality and function benchmarking criteria assessed during NMP. The high discard rate after NMP is attributed to the liberal organ acceptance policy in conjunction with the ability and growing expertise in liver assessment during NMP. The reasons for discard can be found in Supplementary Table 3.

Donor demographics and preservation characteristics are summarized in Table 2. The majority (74%) of the grafts stemmed from DBD donors, the donor age was 59 [15–85] (median and IQR) years and with a BMI of 27.0 [24.0–30.0] kg m^−2^. 26 donors were male, 24 were female. The Donor Risk Index (DRI) was 1.89 [1.63–2.24] and 86% of the organs were retrieved from extended criteria donors.^17^

**Table 2:** Donor demographics and preservation characteristics.

Donor characteristics	Overall cohort (*N* = 50)	Transplanted (*N* = 35)	Discarded (*N* = 15)	*p*-value
Age [y, median (min–max)]	59 (15–85)	62 (26–85)	56 (15–84)	0.7576
Sex ratio [*N* (%)]				
Male	26 (52%)	16 (46%)	10 (67%)	
Female	24 (48%)	19 (54%)	5 (33%)	0.1742
BMI [kg m^−2^, median (IQR)]	27.0 (24.0–30.0)	26.0 (24.0–29.0)	28.0 (23.0–31.0)	0.5018
Cause of death [*N* (%)]				
CVA	29 (58%)	21 (60%)	8 (53%)	
Circulatory	7 (14%)	4 (11%)	3 (20%)	
Trauma	11 (22%)	7 (20%)	4 (27%)	
Other	3 (6%)	3 (8%)	0 (0%)	0.5460
Donor type [*N* (%)]				
DBD	37 (74%)	29 (83%)	8 (53%)	
DCD	13 (26%)	6 (17%)	7 (74%)	0.0292
ECD [*N* (%)]	43 (86%)	28 (80%)	15 (100%)	
DRI [median (IQR)]	1.89 (1.63–2.24)	1.78 (1.61–2.07)	2.22 (1.90–2.38)	0.1471
**Preservation and NMP**				

CIT [min, median (IQR)]	366 (299–447)	349 (299–433)	372 (294–483)	0.7518
Functional WIT^a^ [DCD; min, median (IQR)]	25.50 (21.75–28.75)	26.00 (23.50–33.25)	25.00 (20.75–28.75)	0.8860
Duration of NMP [min, median (IQR)]	882 (574–1277)	824 (603–1177)	1231 (471–1309)	0.7762
Total preservation time [min, median (IQR)]	1240 (983–1576)	1170 (971–1549)	1466 (1106–1686)	0.3629
Bile volume [mL, median (IQR)]	82.0 (7.9–191.8)	185.4 (39.3–348.5	81.9 (18.3–160.0)	0.4385
Lactate clearance [< 2.5 mmol L^−1^ at 2 h NMP, *N* (%)]	44 (88%)	31 (89%)	13 (87%)	
Perfusate lactate [mmol L^−1^, median (IQR)]				
15 min	6.44 (4.22–9.32)	5.55 (3.89–7.33)	7.66 (5.63–8.74)	0.0682
1 h	1.61 (0.94–3.91)	1.22 (0.67–2.44)	3.55 (1.11–5.99)	0.0702
2 h	1.22 (0.78–3.5)	1.11 (0.61–1.89)	1.22 (0.80–3.91)	0.2643
6 h	0.89 (0.44–1.55)	0.78 (0.39–1.22)	2.44 (1.19–2.89)	0.0086
12 h	0.67 (0.36–3.05)	0.56 (0.39–1.16)	2.61 (0.42–3.77)	0.2751
20 h	0.78 (0.44–2.00)	0.72 (0.47–1.19)	2.05 (1.25–4.27)	0.1337
Perfusate AST [U L^−1^, median (IQR)]				
15 min	1631 (730–4097)	1238 (663–2101)	6075 (3655–11246)	0.0005
1 h	1853 (1051–5194)	1482 (729–3136)	7308 (3416–14431)	0.0012
2 h	1918 (991–4430)	1644 (757–3090)	7372 (1941–12606)	0.0070
6 h	2369 (1203–6721)	1820 (1055–4725)	10392 (4297–20842)	0.0010
12 h	5098 (1838–10234)	2547 (1498–6764)	16274 (9006–24193)	0.0009
20 h	7571 (3585–17566)	5507 (2592–8872)	16939 (8745–28965)	0.0797
Perfusate ALT [U L^−1^, median (IQR)]				
15 min	1389 (527–3352)	814 (450–1762)	4672 (2337–11017)	0.0013
1 h	1514 (626–4024)	1065 (549–2067)	6639 (3100–8828)	0.0020
2 h	1561 (631–4416)	1234 (541–2459)	6702 (2382–8535)	0.0037
6 h	1804 (741–5238)	1272 (644–2730)	8448 (3287–14077)	0.0015
12 h	2828 (1071–6667)	1832 (938–3735)	8910 (4796–15045)	0.0025
20 h	4196 (1429–8378)	3136 (1208–4441)	9224 (4425–16903)	0.0295
Perfusate LDH [U L^−1^, median (IQR)]				
15 min	2664 (1243–6960)	2053 (1125–3338)	13017 (3517–23729)	0.0010
1 h	2968 (1671–10891)	2619 (1523–4826)	12957 (6295–26347)	0.0014
2 h	3401 (1556–8640)	2943 (1521–5820)	10475 (4433–21975)	0.0122
6 h	3946 (2095–11115)	3139 (2019–7062)	11908 (5148–25884)	0.0078
12 h	6589 (3394–15502)	4331 (2949–10528)	22103 (9525–37958)	0.0052
20 h	8830 (5270–20269)	6622 (3613–13922)	20917 (8407–27371)	0.0392

a Functional warm ischemia time is applied for DCD donors and defined as the time from a mean arterial pressure below 50 mmHg or an arterial saturation of <80% until the start of cold perfusion.

The median static cold storage was 366 [299–447] min, followed by a NMP time of 882 [574–1277] min, resulting in a total preservation time of 20.66 [16.38–26.27] hours. NMP was uneventful in all cases, lactate was within the normal range at 6 h in all transplanted livers and median total bile production was 82.0 [7.2–191.8] mL. Regular arterial (>150 mL min^−1^) and portal vein (>500 mL min^−1^) blood flows were seen in all liver perfusions. Arterial and inferior vena cava pressures, and *p*_O2_ and *p*_CO2_ values were within physiological range (Supplementary Fig. 2).

**Fig. 2:**
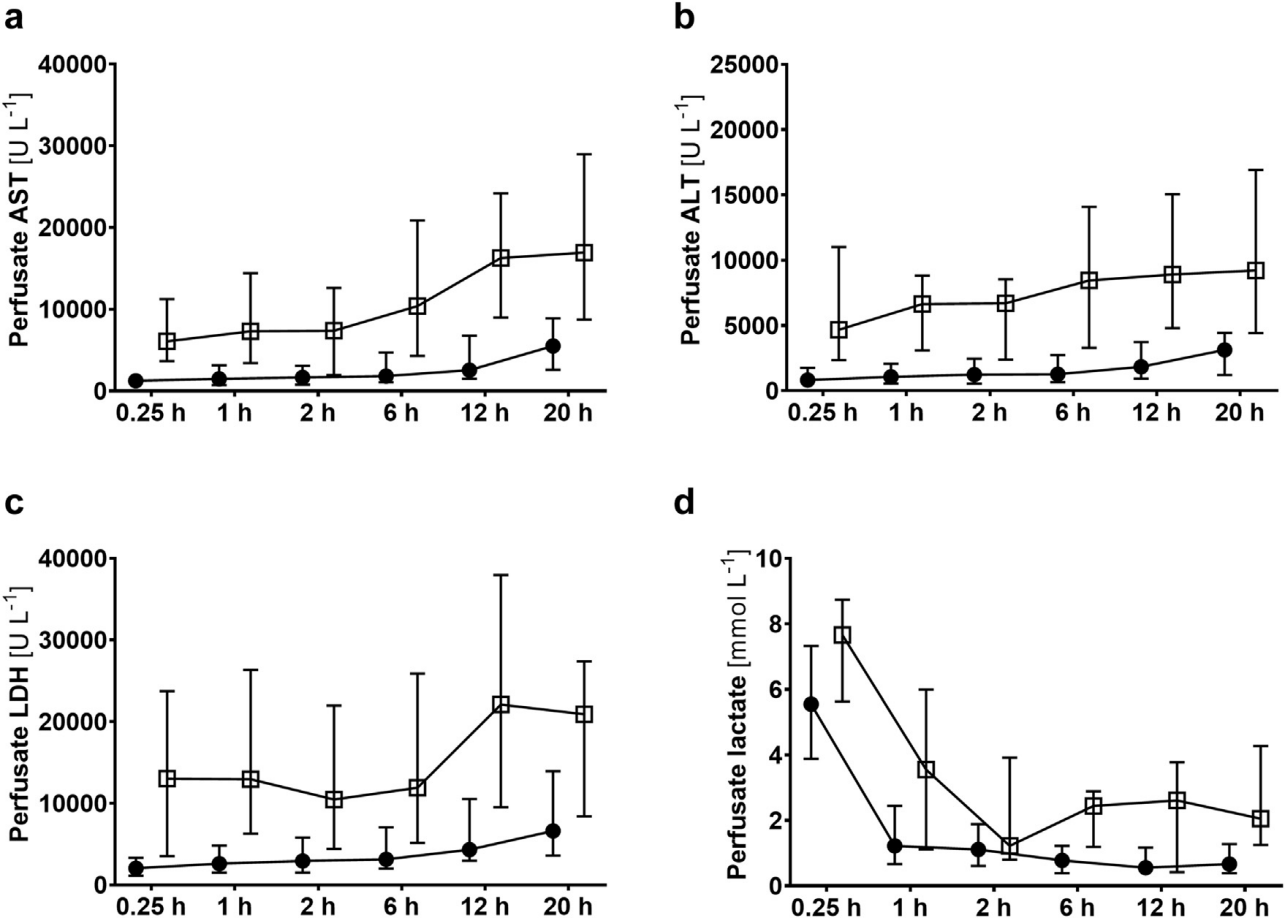
**Perfusate analysis during normothermic machine perfusion.** (a) Aspartate aminotransferase (AST), (b) Alanine aminotransferase (ALT), (c) Lactate dehydrogenase (LDH) activities, and (d) lactate levels for transplanted (solid circles) and discarded (open boxes) livers, expressed as median and interquartile range. For statistical analysis see Table 1.

During NMP, the perfusate was analysed for AST, ALT, LDH and lactate as indicators of organ damage (Fig. 2 and Table 2). Significant differences of transaminases and LDH between transplanted and discarded livers were observed. Interestingly, lactate levels did not show significant differences at 2 h and only approaching significance at 6 h of NMP.

**Fig. 3:**
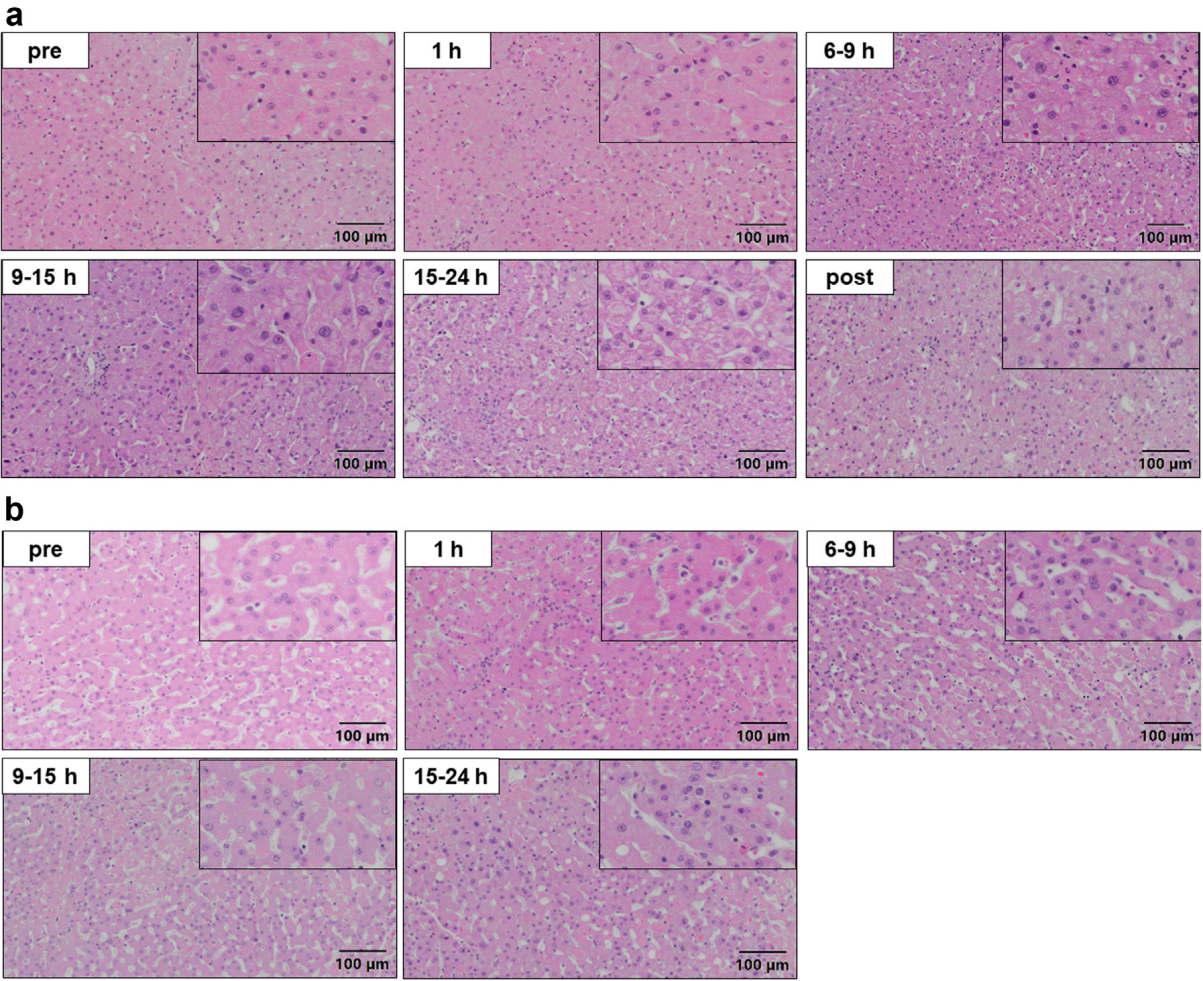
Representative images of tissue biopsies after Haematoxylin & Eosin staining. (a) Livers with subsequent transplantation showed no histopathological alterations in the course of normothermic machine perfusion. (b) Livers discarded after normothermic machine perfusion showed increased signs of necrosis. See also Table S4.

No histopathological alterations during and after NMP were found in livers eventually transplanted (Fig. 3a and Supplementary Table 4). In contrast, aggravated necrosis was observed in the livers discarded after NMP (Fig. 3b). This is further reassuring towards the selection criteria since histology was not considered in the decision process but confirms the difference in liver quality. No induction of apoptosis during NMP was found as indicated by the CCasp3 IHC (Supplementary Table 5, Supplementary Figs. 2 and 3). CD3+ and CD20+ passenger lymphocytes were found in all livers, but no differences between transplanted and discarded livers and no correlation with the outcome was observed. The perfusate analysis of IL-6 display that the inflammatory response is stronger and progressive in discarded livers while no such correlation was observed for TNFα (Supplementary Fig. 4). No downstream effects of inflammation on Kupffer cells (CD68+ cells) or increased TLR4 expression could be found.

Recipient demographics and clinical outcomes are shown in Table 3. The median age was 58 [19–73] years, with a median BMI of 25.50 [17.20–39.90] kg m^−2^ and a MELD score 15.50 [6.00–33.00] at the time of transplantation. Most patients were male (74%). Benchmark cases (according to Muller X et al.^28^) showed a 100% one year patient and 100% one year graft survival. The 90-day overall patient survival rate was 86%. Five patients died because of multi-organ failure due to fasciitis, gastric ulcer perforation and massive bleeding, pancreatitis, colon perforation, aspergillosis, mycosis, and *C. difficile* infection, with *N* = 1 in all cases. The median L-GrAFT score was −0.83 and predictive for patient survival during year one after transplantation (−0.96 *vs.* 0.49).

**Table 3:** Recipient demographics and clinical outcome.

Recipient characteristics	
Age [y, median (min–max)]	58 (19–73)
Sex [*N* (%)]	
Male	26 (74%)
Female	9 (26%)
BMI [kg m^−2^, median (IQR)]	25.50 (17.20–39.90)
MELD at the time of transplantation [median (IQR)]	15.50 (6.00–33.00)
**Outcome**	

1-year patient survival (benchmarking cases)	16 (100%)
1-year graft survival (benchmarking cases)	16 (100%)
1-year patient survival (total)	28 (80%)
1-year graft survival (total)	26 (74%)
L-GrAFT [median (IQR)]	−0.83 (−2.39–3.13)
MEAF [median (IQR)]	4.67 (1.86–8.9)
EAD [*N* (%)]	13 (37%)

### Dynamics of mitochondrial respiration during normothermic machine perfusion

**Fig. 4:**
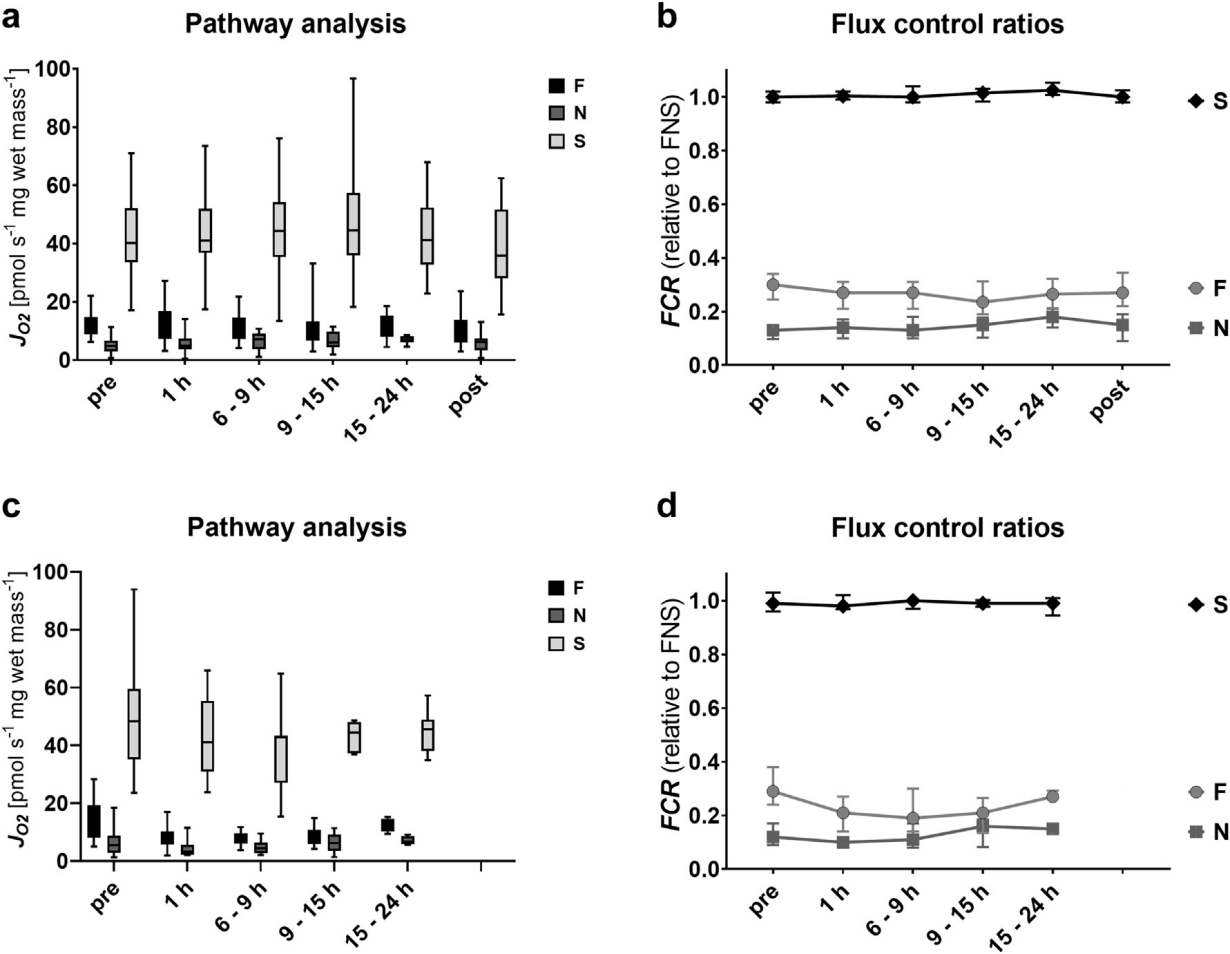
Pathway control of mitochondrial respiration after static cold storage, during normothermic machine perfusion, and upon reperfusion. Respiration in the OXPHOS state with saturating ADP concentrations (5 mM). Octanoylcarnitine (0.5 mM) and malate (0.1 mM) were added as substrates for the fatty acid oxidation (F)-linked pathway; pyruvate (5 mM), malate (2 mM) and glutamate (10 mM) as substrates for the NADH (N)-linked pathway or rotenone (0.5 μM) and succinate (10 mM) for the S-linked pathway in subsequently transplanted (a) and discarded livers (c). Relative contributions of the three substrate pathways, expressed as flux control ratios (*FCR*) in transplanted (b) and discarded livers (d). The *FCR* of the F-, N-, and S-linked pathways were calculated relative to the maximum OXPHOS capacity reached after addition of substrates of all three pathways. In the box plots, data are represented as median, interquartile range and min–max values while in (b) and (d) median and interquartile ranges are shown. No significant changes were found between the transplanted and discarded livers (*p* > 0.05). #*p* < 0.05 compared to pre values within groups (two-way ANOVA).

As a first step, we analysed the capacity of mitochondrial oxidative phosphorylation (OXPHOS) in crude homogenates from liver biopsies for the NADH-, FAO- and succinate-linked pathways (N_*P*_, F_*P*_, S_*P*_; Fig. 4a and Table 4). In biopsies from static cold storage (SCS) livers, wet-mass-specific respiration was highest for the S_*P*_ (40.23 [33.57–52.15] pmol s^−1^ mg^−1^), with large variation between grafts. In contrast, respiration was markedly lower for the N*_P_* and F_*P*_ (4.81 [2.99–6.67] pmol s^−1^ mg^−1^ and 12.54 [8.88–14.82] pmol s^−1^ mg^−1^, respectively). After start of NMP, OXPHOS capacity remained stable in all three pathways, indicating no further damage to the mitochondrial electron transfer and phosphorylation systems as a response to reperfusion. Moreover, in samples taken after reperfusion upon transplantation, no significant decrease in respiration was detected. Next, we calculated the relative contribution of single substrate pathways as *FCR* relative to the maximum OXPHOS capacity reached with all substrates. As shown in Fig. 4b, succinate-linked respiration alone was sufficient to saturate OXPHOS capacity, with no control of the S-pathway over respiration, expressed as an *FCR* = 1.0. This pattern of pathway control reflects incomplete additivity.^21^ While the *FCR* of the S-pathways remains stable during SCS, NMP and reperfusion, slight changes of the *FCR* in the F- and N-pathways were detected. During NMP, the contribution of the F-pathway decreases slightly, reaching significance after 9–15 h. In parallel, the N-pathway slowly increases, reaching a peak in samples taken 15–24 h after start of NMP. Quite importantly, the *FCR* of these pathways returns to baseline after reperfusion. Similar patterns were observed in discarded grafts (Figs 4c, d).

**Table 4:** Mitochondrial respiration in the OXPHOS state and corresponding flux control ratios.

Transplanted	pre	1 h	6–9 h	9–15 h	15–24 h	post
F-pathway [pmol s^−1^ mg^−1^]	12.54 (8.88–14.82)	11.18 (7.26–16.83)	10.83 (7.23–14.60)	11.48 (6.71–13.28)	10.48 (7.97–15.20)	10.72 (6.07–13.77)
N-pathway [pmol s^−1^ mg^−1^]	4.81 (2.99–6.67)	4.91 (3.66–7.43)	7.19 (3.75–9.10)	6.06 (4.37–9.76)	6.82 (6.19–8.13)	6.14 (3.44–7.49)
S-pathway [pmol s^−1^ mg^−1^]	40.23 (33.57–52.15)	41.04 (36.83–51.96)	44.39 (35.38–54.13)	44.47 (35.9–57.39)	41.17 (32.74–52.35)	35.89 (28.07–51.70)
F *FCR*	0.30 (0.25–0.34)	0.27 (0.21–0.31)	0.27 (0.21–0.31)	0.24 (0.19–0.31)	0.27 (0.21–0.32)	0.27 (0.22–0.35)
N *FCR*	0.13 (0.10–0.15)	0.14 (0.10–0.17)	0.13 (0.10–0.18)	0.15 (0.10–0.18)	0.18 (0.14–0.21)	0.15 (0.09–0.19)
S *FCR*	1.00 (0.98–1.02)	1.00 (0.99–1.02)	1.00 (0.98–1.04)	1.01 (0.98–1.03)	1.02 (1.01–1.05)	1.00 (0.98–0.13)

Discarded	pre	1 h	6–9 h	9–15 h	15–24 h
F-pathway [pmol s^−1^ mg^−1^]	13.81 (8.09–19.24)	7.68 (5.71–10.27)	7.78 (6.10–9.51)	9.08 (5.93–10.76)	12.42 (10.26–14.58)
N-pathway [pmol s^−1^ mg^−1^]	5.50 (2.82–8.76)	3.20 (2.11–5.54)	4.37 (2.71–6.31)	6.18 (3.54–9.13)	6.68 (5.78–8.60)
S-pathway [pmol s^−1^ mg^−1^]	48.26 (35.15–59.53)	41.05 (30.97–55.36)	42.74 (27.01–43.31)	44.44 (37.29–47.98)	45.56 (38.02–48.92)
F *FCR*	0.29 (0.24–0.38)	0.21 (0.14–0.27)	0.19 (0.14–0.30)	0.21 (0.14–0.27)	0.27 (0.25–0.29)
N *FCR*	0.12 (0.09–0.17)	0.10 (0.08–0.11)	0.11 (0.08–0.17)	0.16 (0.08–0.19)	0.15 (0.14–0.16
S *FCR*	0.99 (0.96–1.03)	0.98 (0.97–1.02)	1.00 (0.97–1.00)	0.99 (0.98–1.00)	0.99 (0.95–1.01)

Respiration in the OXPHOS state with saturating ADP concentrations (5 mM) normalized for wet tissue mass. Octanoylcarnitine (0.5 mM) and malate (0.1 mM) were added as substrates for fatty acid oxidation (F-pathway); pyruvate (5 mM), malate (2 mM) and glutamate (10 mM) as substrates for the NADH-linked N-pathway; or rotenone (0.5 μM) and succinate (10 mM) for the S-pathway. Relative contributions of the three electron transfer pathways are expressed as flux control ratios (*FCR*). The *FCR* of the F-, N-, and S-pathways were calculated relative to the maximum OXPHOS capacity reached after addition of substrates and ADP for all three pathways. Results are shown as median (interquartile range).

### In-depth analysis of succinate-linked mitochondrial respiration

#### S-linked respiration during SCS

**Fig. 5:**
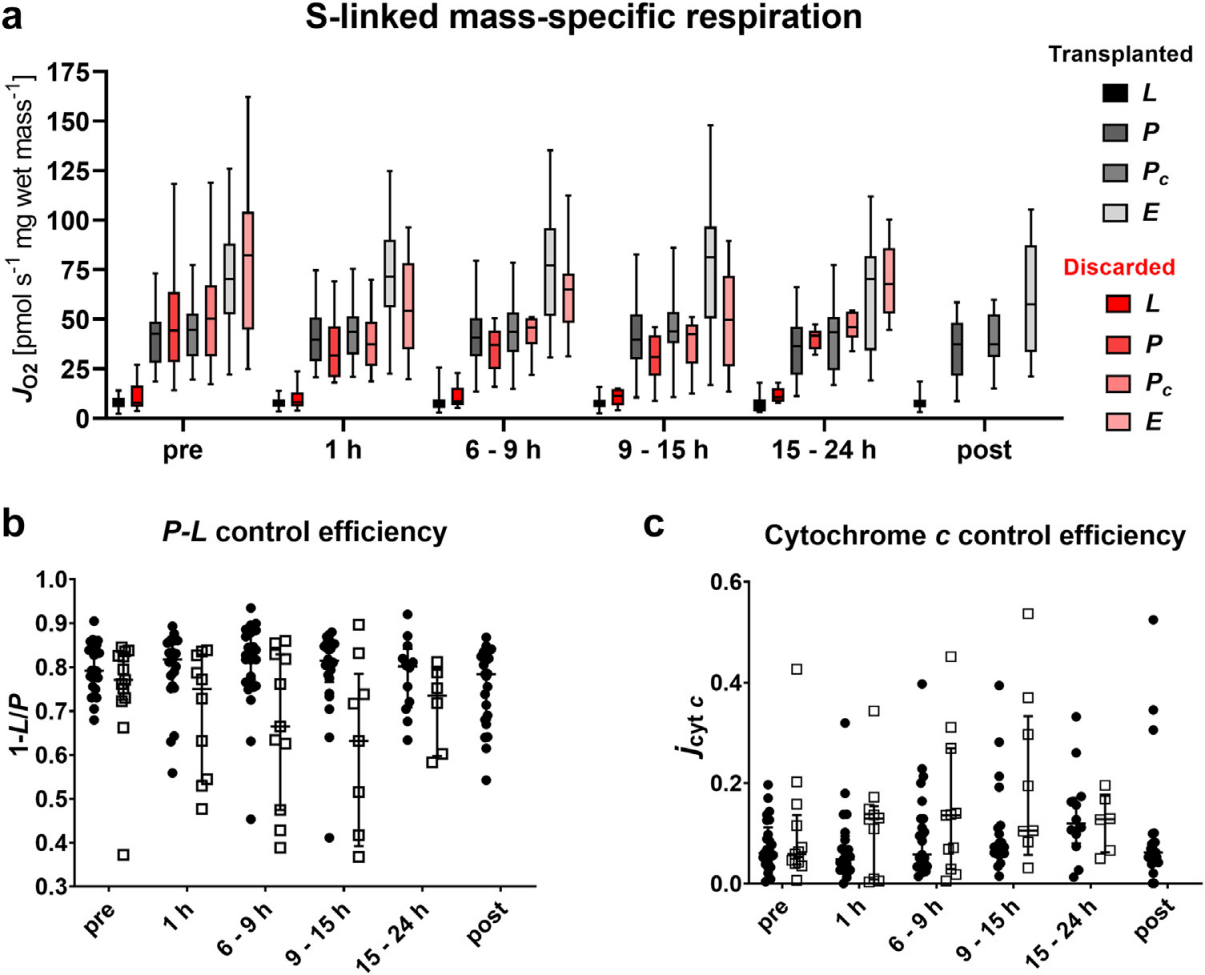
Effect of normothermic machine perfusion on bioenergetic function. (a) Analysis of different respiratory coupling states: LEAK (*L*) respiration was reached after addition of succinate (10 mM) as substrate for the S-linked respiration and rotenone (0.5 μM) for Complex I inhibition in the absence of ADP. Then, ADP (5 mM) was added to analyse OXPHOS capacity (*P*) and subsequent addition of cytochrome *c* (10 μM) allowed to evaluate integrity of the mitochondrial outer membrane (*P*_c_). Carbonyl cyanide p-trifluoromethoxyphenyl hydrazone (CCCP) was titrated step-wise until maximum respiration to assess the electron transfer (*E*) capacity. (b) For calculation of the *P*-*L* control efficiency, S-linked LEAK respiration served as background state and OXPHOS capacity served as reference state (1-*L*/*P*), which allows for evaluation of the efficiency of ATP production. (c) Cytochrome *c* control efficiency was calculated with OXPHOS capacity as background state and OXPHOS capacity upon addition of cytochrome *c* (*P*_c_) as reference state (1-*P*/*P*_c_), as a measure of mitochondrial outer membrane integrity. In the box plots, data are represented as median, interquartile range and min–max values while in the scatter plots single datapoints, median and interquartile range are shown. (b + c) Solid circles represent the transplanted, open boxes represent discarded livers. No significant differences within the coupling states or between the transplanted and discarded livers were found (*p* > 0.05, two-way ANOVA).

A detailed analysis of the mitochondrial function and coupling control efficiencies was performed for the S-linked pathway, since this pathway alone could saturate the OXPHOS capacity (Fig. 5). At the end of SCS, we observed a mean S-linked OXPHOS capacity S_*P*_ of 40.49 [31.34–50.59] pmol s^−1^ mg^−1^ (Fig. 5a). The addition of cytochrome *c* did lead to a merely slight increase in respiration (43.48 [33.31–53.36] pmol s^−1^ mg^−1^), reflected by a low cytochrome *c* control efficiency (0.06) (Fig. 5c). This finding, together with low LEAK respiration S_*L*_ (8.21 pmol s^−1^ mg^−1^) and a *P-L* control efficiency with a mean value of 0.8 (Fig. 5b) indicate no extensive damage to the mtOM or mitochondrial inner membrane (mtIM), respectively. Stepwise titration of CCCP resulted in a mean ET capacity S_*E*_ of 70.33 pmol s^−1^ mg^−1^ in the measurements at the end of SCS. Thus, ET capacity surpasses OXPHOS capacity, resulting in a *E-P* control efficiency 1-*P*_*c*_*/E* of 0.40, indicating a strong control of the phosphorylation system over ET capacity.

#### Coupling control of the S-pathway remains stable during NMP

**Fig. 6:**
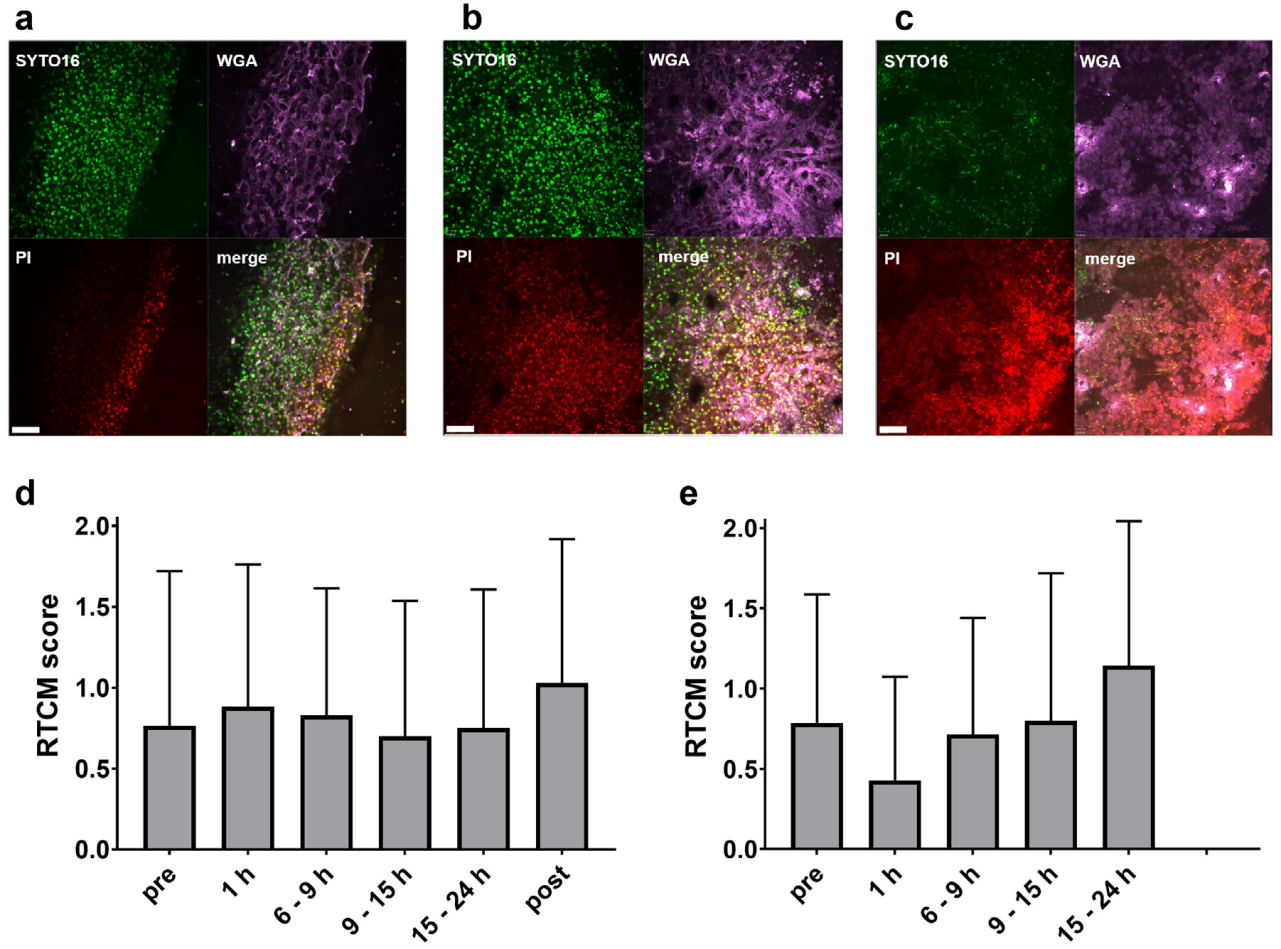
Real-time live confocal microscopy during normothermic machine perfusion. (a–c) Representative RTCM images stained with SYTO 16 (green), PI (red), WGA (pink) and merge; score 0 = highly viable cells (a), score 1 = number of viable and dead cells equals (b), score 2 = highly non-viable cells (c). Scale bars represent 100 μm. Semiquantitative scoring for the transplanted (d) and discarded livers (e). Results are expressed as mean ± SD.

It remains unclear to date, if reperfusion under the conditions of NMP is benign and less damaging when compared to a reperfusion in a patient. In our study, mitochondrial respiration did not deteriorate during NMP compared to the “pre” measurements (Fig. 5). Mass-specific OXPHOS, LEAK and ET respiration remained stable during NMP and transplantation (Fig. 5a). The considerable variability between grafts also seems to be a robust description of the bioenergetic status since no significant changes were detected throughout the course of NMP. Respiration also remained stable in cases with prolonged NMP up to 24 h. These findings agreed with the results of the RTCM analysis (Fig. 6). In biopsies taken at the end of SCS, a mean RTCM score of 0.765 was observed. A modest deterioration at 1 h and 6–9 h of NMP (0.882 and 0.829), followed by a stabilization/improvement in the further course of NMP was found. The mean RTCM score rose to 1.029 after reperfusion, but this increase did not reach statistical significance.

**Fig. 7:**
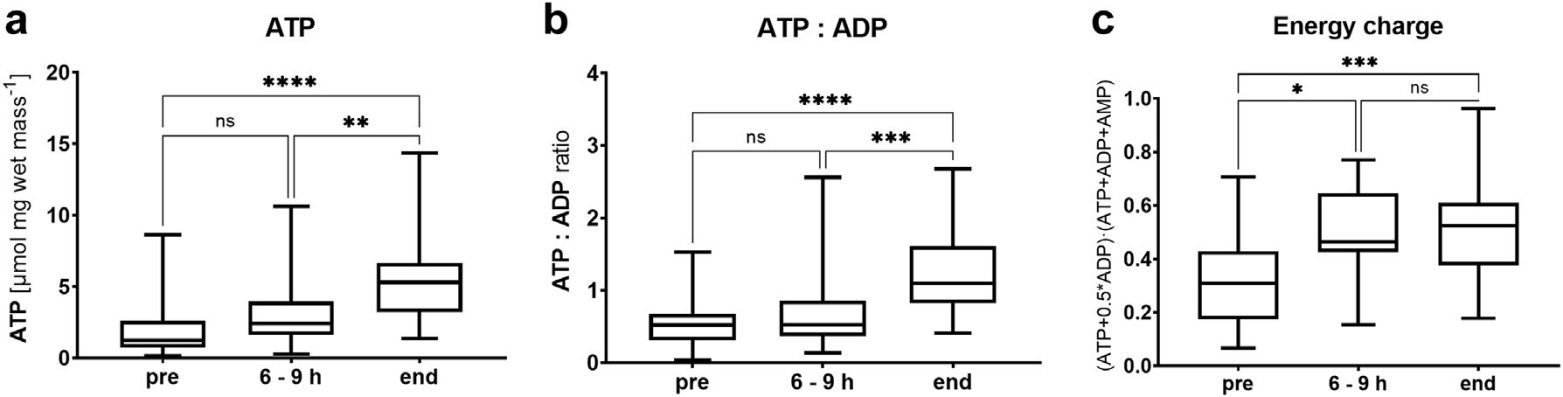
Analysis of adenine nucleotides in tissue biopsies during normothermic machine perfusion of transplanted livers. (a) ATP concentration in liver biopsies. (b) Ratio of ATP to ADP. (c) Energy charge calculated as (ATP + 0.5∗ADP)/(ATP + ADP + AMP). Results are represented as median, interquartile range and min–max values. (∗*p* < 0.05, ∗∗*p* < 0.01, ∗∗∗*p* < 0.001, ∗∗∗∗*p* < 0.0001, ns = no significance; comparisons between time points, Kruskal–Wallis test).

Assessment of the *quality* of the mitochondrial respiration revealed an unchanged mean *P-L* control efficiency during NMP (0.8) (Fig. 5b). The low cytochrome *c* control efficiency also remained stable in most cases (Fig. 5c). Nevertheless, single grafts exhibited *P-L* control efficiencies as low as 0.55. Furthermore, we found a slight increase in the *P-L* control efficiency in samples taken after reperfusion. ATP tissue levels significantly increased during machine perfusion (*p <* 0.0001) (Fig. 7). Moreover, we observed significant changes for the ATP:ADP and energy charge ratio (*p <* 0.0001 and *p* = 0.0003, respectively). Similar trends were found for the discarded group (Supplementary Fig. 5). Importantly*, P-L* control efficiency reflects the isolated mitochondrial ATP production capacity while tissue adenylate levels displays the net cellular ATP production and consumption.

**Fig. 8:**
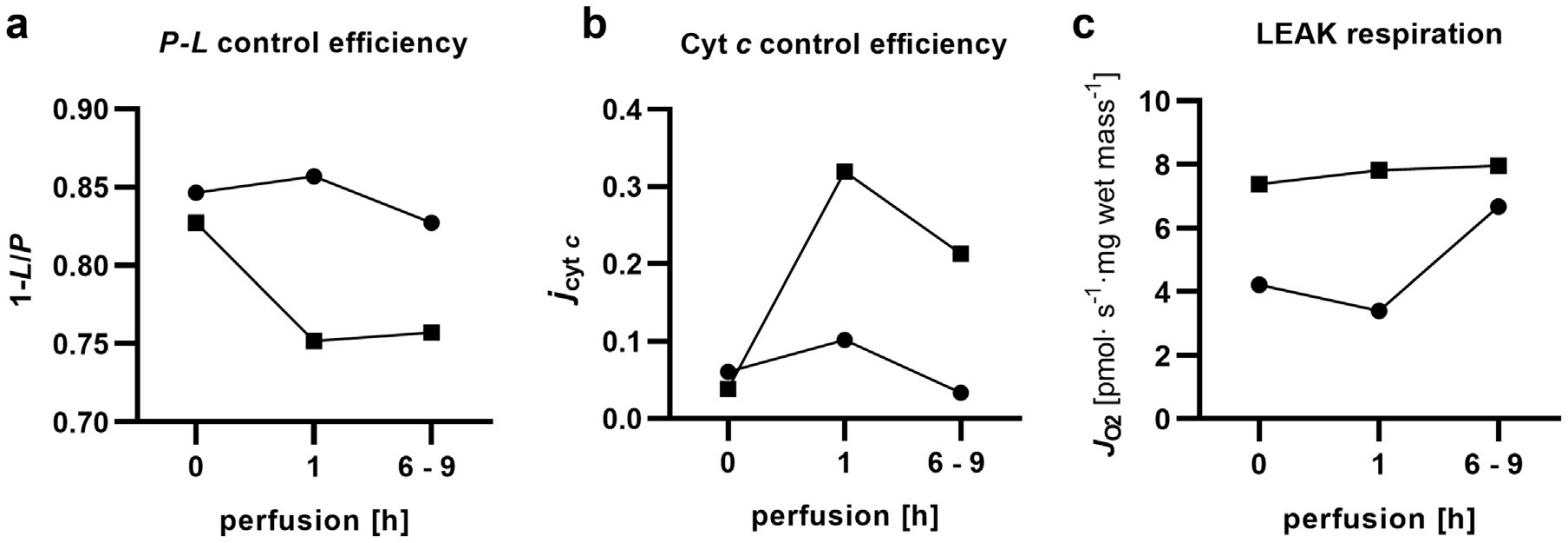
Bioenergetic function before and during NMP in good and poor performing livers. Two examples are depicted representing good performance (solid circles) and poor performance (open boxes) for the mitochondrial parameters *P*-*L* control efficiency (a), cytochrome *c* control effienicy (b) and LEAK respiration (c).

Some livers seem to suffer from a reperfusion injury after the initiation of NMP. This is also indicated by severely elevated cytochrome *c* control efficiencies of up to 0.4 (Fig. 8). In such cases, cytochrome *c* efficiency remained high after transplantation, while the remaining grafts did not express mtOM damage after reperfusion. The injury phenotype was consistent with an elevated RTCM score of 2 (Fig. 6c). In contrast, livers in the discarded cohort expressed lower *P*-*L* control efficiencies and slightly elevated cytochrome *c* control efficiencies during NMP as compared to the transplanted cohort. Since the study was not powered to achieve this, the difference did not reach statistical significance.

Liver mitochondrial respiration exhibited a relative robustness and stability over time (RM ANOVA). We did not find significant differences between transplanted and discarded livers with respect to the dynamics of mitochondrial function. However, some time-dependent changes during NMP and a considerable variability in most mitochondrial parameters between liver grafts were observed. While this requires further investigations, the overall relative stability together with the high variability of the liver's bioenergetic condition suggests that further prolonged preservation may allow to differentiate between livers eventually recovering *vs.* livers with progressive injury.

### Mitochondrial integrity during NMP

**Fig. 9:**
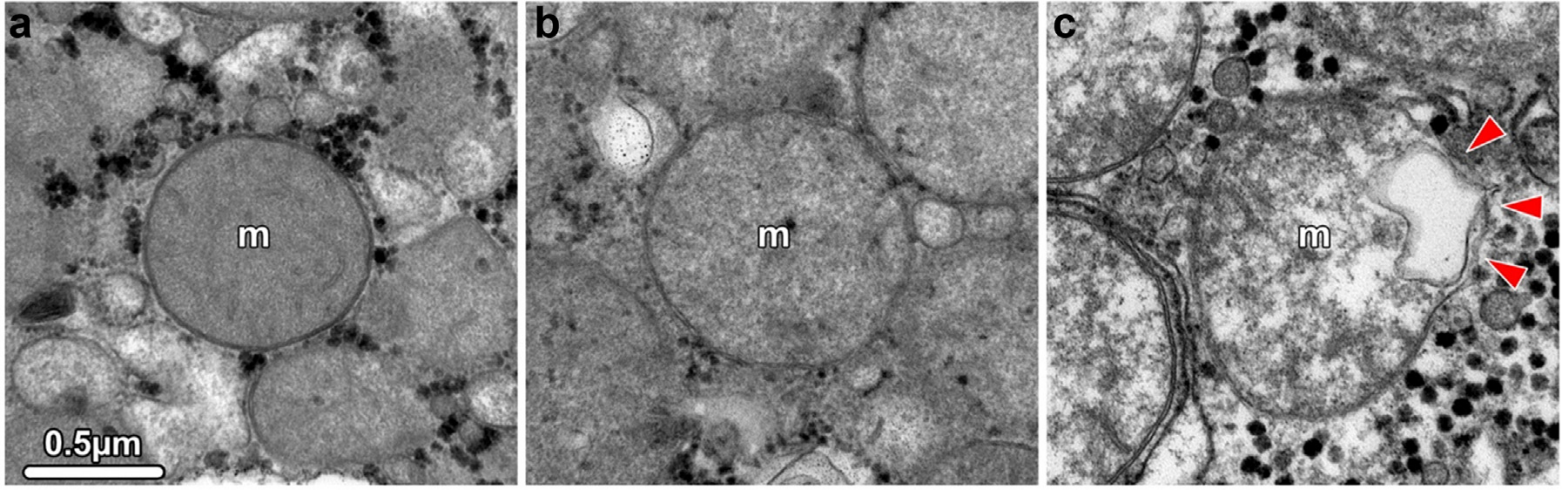
Transmission electron micrographs of mitochondria showing structural changes in hepatocytes. (a) Normal mitochondria showing an intact outer and inner membrane. The inner membrane is differentiated into the cristae, which extend deep into the electron-dense matrix. (b, c) Altered mitochondria show loss of cristae, an electron-translucent and swollen matrix, and rupture of the outer membrane (arrowheads in c). All images have the same magnification.

**Fig. 10:**
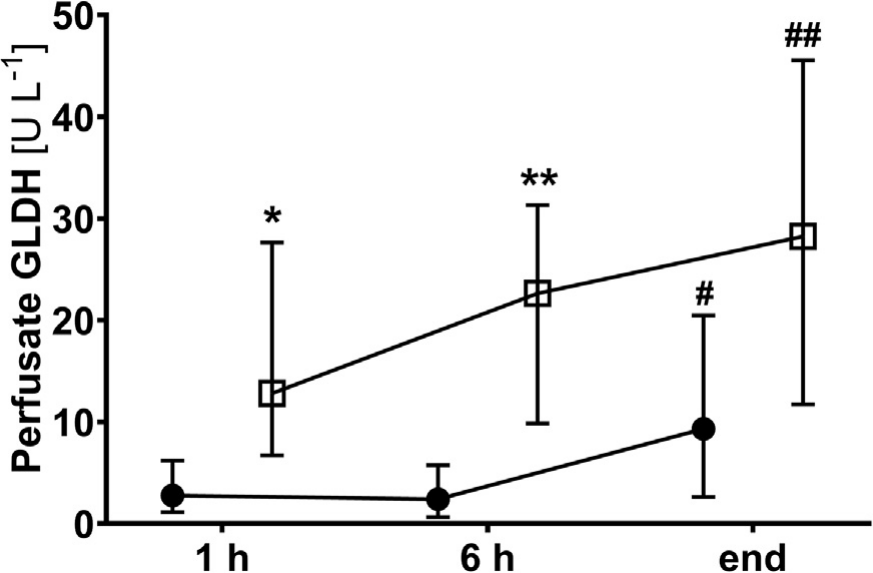
Perfusate GLDH levels for transplanted (solid circles) and discarded (open boxes) livers. Values are expressed as median and interquartile range (∗*p* < 0.05, ∗∗*p* < 0.01, transplanted *vs.* not transplanted group; #*p* < 0.05, ##*p* < 0.01, compared to 1 h values within groups; two-way ANOVA).

Heterogeneity of the mitochondrial morphology of hepatocytes was confirmed at the ultrastructural level using TEM assessment in selected livers. While the mtOM was found intact in most cases, the typical architecture of the mtIM folded into cristae and was lost in damaged mitochondria. In such cases, a swollen and electron-translucent matrix was observed (Fig. 9a–c). Moreover, the analysis of GLDH as a mitochondrial marker enzyme released into the perfusate due to damage confirmed these findings (Fig. 10). No increase of GLDH release during the first 6 h of perfusion was found and levels remained in a physiological range throughout NMP. In contrast, the perfusate GLDH activity was significantly higher in discarded livers.

### Predictive value of mitochondrial respiratory function on clinical outcome

In the next step, we investigated whether the S-pathway measurements including LEAK respiration, OXPHOS capacity, OXPHOS capacity after cytochrome *c* addition, ET capacity, and control efficiencies of respiration have predictive value towards the postoperative outcome. We analysed these parameters at single time points and as an integral of the perfusion time.

**Fig. 11:**
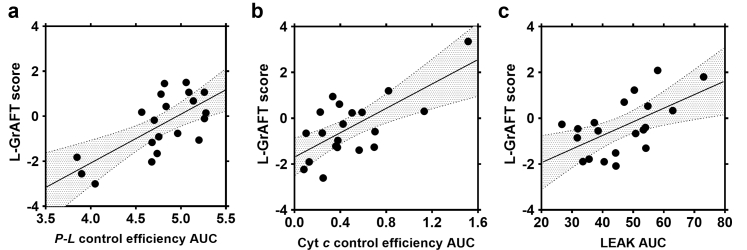
Partial residual plots of mitochondrial biomarkers correlating with L-GrAFT score in the multivariate regression model. (a) Area under the curve (AUC) of *P*-*L* control efficiency, beta value 0.73; (b) AUC of cytochrome *c* control efficiency, beta value −1.79, and (c) AUC of LEAK respiration, beta value −0.35. Solid dots represent partial residuals, the solid line the regression line of the multivariate model and the 95% confidence intervals are marked in grey.

A stepwise algorithm was used to test the associations of possible predictors with the outcome as defined by L-GrAFT. No correlations for any of the individual time point measurements were found (Supplementary Table 6), but trends for some parameters were observed. Cytochrome *c* control efficiency in pre and 1 h biopsies reached *p*-values of 0.295 and 0.229, respectively. In line with this, the *P-L* control efficiency at these two time points showed similar trends (0.290 and 0.324). Since we observed significant dynamics during the course of liver NMP in single cases, we calculated the AUC for S-pathway related parameters to include the time-component of NMP. Using a stepwise algorithm, we identified three different mitochondrial parameters, which predict the clinical outcome. A significance or trend towards significance was found for type II ANOVA (*F*(4,15) = 2.54, *p* = 0.83) towards L-GrAFT (Table 5), but not towards MEAF or EAD (Supplementary Tables 7 and 8, respectively) for the following parameters: (i) A low AUC LEAK respiration correlated with a good clinical performance after transplantation (*p* = 0.141). (ii) AUC cytochrome *c* control efficiency (*p* = 0.021), a mitochondrial parameter which allows to evaluate the damage to the mtOM. In line with AUC LEAK, the correlation indicates that the preserved integrity of the mtOM predicts a better clinical outcome. Further, we found a positive interaction of parameters (i) and (ii) (*p* = 0.131). (iii) Interestingly, there was a strong negative correlation for the efficacy of mitochondrial ATP production with the clinical outcome (*p* = 0.026). A partial residual plot of those parameters is shown in Fig. 11.

**Table 5:** ANOVA table (Type II tests).

Parameter	b	conf	beta	*p*-value
1-*L*/*P* AUC	−10.3	[1.64, −22.30]	0.73	0.026
*j*_cyt__*c*_ AUC	2.17	[4.04, 0.30]	−1.79	0.021
LEAK AUC	−6.47	[5.38, −18.30]	−0.35	0.141
*j*_cyt__*c*_ AUC: LEAK AUC	−0.0375	[0.09, −0.16]	2.55	0.131
Residuals	0.198	[0.46, −0.06]		

*r*^2^ = 0.40, ad.*r*^2^ = 0.25, *F*_(4, 15)_ = 2.54, *p* = 0.083.

## Discussion

In the present study, we analysed mitochondrial performance, tissue viability and structural mitochondrial damage in liver biopsies undergoing NMP. We demonstrated that mitochondrial respiration is maintained during NMP. Further, we found a correlation of mitochondrial coupling and mtOM damage markers with the clinical outcome, suggesting these as biomarkers for machine perfused organs.

One of the major advantages of NMP is the preservation of donor organs under close-to-physiological conditions. This enables real-time analysis of metabolic and biosynthetic functions prior to transplantation and may help to improve the decision-making process for the selection of optimal organs. For example, Mergental et al. reported successful transplantation of 22 organs which were initially declined based on lactate clearance to ≤2.5 mmol L^−1^ within 4 h of NMP.^13^ In addition to lactate clearance, several parameters which may predict the clinical outcome have been proposed, including perfusate enzyme levels and pH of the secreted bile.^11^^,^^29^ However, the majority of the assessment tools and biomarkers are injury markers and metabolites from the perfusate. While single biomarkers have proven useful, a multidimensional assessment of organ quality, viability, and function is desirable and may aid in the decision-making. In the present study, we assessed the bioenergetic function and viability of the liver tissue by mitochondrial respiration analysis and confocal imaging.

Intact mitochondria are indispensable as the most efficient sources covering the energy demand of the liver by the aerobic respiration. Convergent substrate pathways fuel the electron transfer system and drive ATP production by coupled processes termed collectively as oxidative phosphorylation OXPHOS.^1^^,^^30^ Due to lack of oxygen during and after organ procurement, OXPHOS is inhibited, leading to loss of intracellular ATP. Upon reperfusion, extensive ROS production in various intra- and extracellular compartments aggravate damage to membrane lipids and proteins. These processes severely impact the mitochondrial membranes, their embedded enzyme complexes and linked enzymes.^5^^,^^31^ As targets of donor-related (pre-existing) damage and secondary injuries, mitochondria are potential candidates for the assessment of organ quality and function. This prompted us to perform an in-depth assessment of the bioenergetic function of machine perfused livers using HRR by directly measuring mitochondrial respiration in serial tissue biopsies in a large set of machine perfused livers. A highly diverse mitochondrial respiration was found at the end of SCS. Since bioenergetic performance during SCS did not correlate with the clinical outcome, we suspect reversibility of the bioenergetic state upon reperfusion. In fact, upon initiation of NMP, metabolic activity recovered to an individual steady state in all transplanted livers. No changes were observed in the substrate pathway control or in the overall mass-specific mitochondrial respiration.

We found the succinate-pathway to be the prominent mitochondrial substrate. Succinate saturates mitochondrial OXPHOS respiration during SCS. Contribution of the NADH- and FAO-pathways are considerably lower, but significant. This is in line with previous reports where the dominance of the succinate pathway was shown in isolated mouse^32^^,^^33^ and human^3^ mitochondria and in tissue homogenates of the porcine liver.^34^

Damage to the mtOM leads to release of cytochrome *c* as reflected by an impaired mitochondrial function that is partially recovered after exogenous cytochrome *c* supplementation.^16^ As demonstrated by the cytochrome *c* control factor assessment, mtOM stabilized under NMP. This agrees with the outcome from the RTCM and GLDH analysis showing no decrease in cell viability and mitochondrial integrity during the first 6 h of perfusion in livers deemed suitable for transplantation.

Respiration in the presence of substrates but in absence of ADP displays LEAK respiration. Respiration in this dissipative state only serves to compensate for the “leakiness” of the mtIM. This state is a major factor in the uncoupling of substrate oxidation and ATP production. Thus, we calculated the *P*-*L* control efficiency, which is the normalized ratio of respiration in the LEAK state compared to the OXPHOS state. This parameter allows to evaluate the coupling efficiency of mitochondria.^21^ Both, cytochrome *c* control efficiency and LEAK respiration predicted the clinical outcome after transplantation. The degree of damage closely correlated with L-GrAFT. *P*-*L* control efficiencies reveal that the majority of livers are bioenergetically competent. Interestingly, a lower *P*-*L* control efficiency in livers during the early perfusion predicted a better clinical outcome. This mild mitochondrial uncoupling at the beginning of reperfusion may be a protective mechanism during IRI.^35^

The stability of mitochondrial respiration during NMP and the predictive value of the integrated parameters during the early NMP phase (up to 6 h) are encouraging. This may help to further advance the search of novel biomarkers and serve the decision-making process during NMP.^36^ While respiration during SCS has limited predictive value, the restoration of the bioenergetic state during NMP and the relative stability of respiration during NMP indicates that a single assessment of mitochondrial function at any point in time displays the true bioenergetic capacity of a liver. In contrast, the energy charge and tissue ATP levels depend on both the ATP production by mitochondrial OXPHOS and the consumption through cellular metabolism.

The decision for selecting organs of sufficiently good quality for transplantation in this study was based on previously published criteria including injury markers, such are perfusate transaminase levels and lactate clearance. As a logical consequence of the fact that those criteria were applied in the selection process, discarded livers express significantly worse values. However, the robustness of these benchmarking criteria as biomarkers with predictive capacity are limited. Ergo, some of the livers have shown suboptimal function after transplantation and some discarded liver may have functioned well if transplanted. The observation that mitochondrial respiration did not differ significantly between transplanted and discarded organs while HRR data during NMP were predictive of the outcome suggests that there is added value of HRR data in a currently imperfect benchmarking process.

There are obvious limitations of the study. (i) It is a single-centre study and confirmation of our findings are required. (ii) High-resolution respirometry is a sensitive, but also technically advanced method. Inter-user variability and technical variability needs to be ruled out by performing technical and biological replicates, strict standardization of the technique and quality control measures.^37^ The robustness of the predictive capacity of some HRR measures requires further attention prior to its potential implementation as a biomarker. (iii) The relative heterogeneity of human livers retrieved for transplantation is substantial. Pre-existing conditions may impact on the bioenergetic capacity of an organ and alter the results. (iv) The decision to transplant or discard a liver were based on established, but not formally approved benchmarking criteria. Data on the transplant outcome of discarded livers are missing and no correlation with their bioenergetic performance was possible.

This trial delivers evidence that bioenergetic function of livers during NMP does not deteriorate during perfusion times of up to 24 h. HRR may add to the armamentarium in *ex vivo* liver quality assessment and serve as a biomarker for the outcome.

## Contributors

Conceptualization (AM, JH, TH, SS), data curation (AM, JH, MLB, TDM, FN, MB, MF, MH, BZ, GO, MS), data analysis (AM, JH), data verification (SS, TH), supervision (SS), writing – original draft (AM, JH, TH, SS), writing – review & editing (AM, JH, MLB, BC, TDM, FN, MB, MF, MH, AW, RO, TR, JT, DÖ, HZ, HT, EG, TH, SS). All authors read and approved the final manuscript. SS was responsible for the decision to submit the manuscript.

## Data sharing statement

Data supporting the figures and tables of this manuscript (deidentified patient and analysis data) are available from the corresponding author upon reasonable request.

## Declaration of interests

SS received grants, consulting fees or honoraria for lectures from the following entities, not related to the present study: Novartis, Sandoz, Bridge to Life, Chiesi, Neovii, Organ Recovery, Astellas, Teva, Merck, Atara, NefroHealth, ITB, BMS, Sanofi, OrganOx. EG is founder and CEO of Oroboros Instruments. The other authors have no potential conflict of interest to report.
