# Supplementary material for: Mitochondrial respiration during normothermic liver machine perfusion predicts clinical outcome
Source: eBioMedicine. 2022 Oct 29;85:104311. doi: 10.1016/j.ebiom.2022.104311 (PMC9626552; doi:10.1016/j.ebiom.2022.104311)
Supplement: Multimedia component 1 [file mmc1.pdf]

Supplementary material

Table of contents:

Table S 1: SUIT01 protocol……………………………………………………………………1
Table S 2: SUIT02 protocol……………………………………………………………………1
Table S 3: Reasons for discard of the non-transplanted livers…………………………...2
Figure S 1: Machine perfusion parameters during NMP…………………………………2
Table S 4: Histological assessment……………………………………………………….3
Table S 5: Immunohistochemistry…………………………...……………………..…3
Figure S 2: Immunohistochemistry of transplanted livers……………….…………………4
Figure S 3: Immunohistochemistry of discarded livers……………..……………………5
Figure S 4: Perfusate analysis of IL-6 and TNFα……………………...…………………..6
Figure S 5: Analysis of adenine nucleotides……………………………………………..6
Table S 6: ANOVA Table (Type II tests)……………………………………………………..7
Table S 7: ANOVA Table (Type II tests) – MEAF……...…………………………………7
Table S 8: ANOVA Table (Type II tests) – EAD……………………………………………..7

Table S 1: SUIT01 protocol

Step	Compound	Concentration	Coupling control state	ET-pathway state	Function
1	Tissue homogenate (2 mL)	1 mg wet mass·mL^{-1}		ROX	
2	ADP-Mg^{2+} (Merck, 117105)	5 mM		ROX	Substrate of ATP synthase
3	Malate (Sigma, M1000)	0.1 mM			NADH-linked substrate
4	Octanoylcarnitine (APExBIO, B6371)	0.5 mM	OXPHOS (*P*)	FAO	Substrate for fatty acid oxidation (FAO)
5	Cytochrome *c* (Sigma, C7752)	10 µM	OXPHOS (P_c)	FAO	Evaluate outer mt-membrane integrity
6	Pyruvate (Sigma, P2256)	5 mM	OXPHOS (P_c)	FAO+NADH	Feeds TCA (via Acetyl-CoA)
7	Malate (Sigma, M1000)	2 mM	OXPHOS (P_c)	FAO+NADH	NADH-linked substrate
8	Glutamate (Sigma, G1626)	10 mM	OXPHOS (P_c)	FAO+NADH	NADH-linked substrate
9	Succinate (Sigma, S2378)	10 mM	OXPHOS (P_c)	FAO+NADH+S	Substrate of Complex II
10	Rotenone (Sigma, R8875)	0.5 µM	OXPHOS (P_c)	S	Inhibition of Complex I
11	Antimycin A (Sigma, A8674)	2.5 µM		ROX	Inhibition of Complex III

Table S 2: SUIT02 protocol

Step	Chemical	Concentration	Coupling control state	Function
1	Tissue homogenate (2 mL)	1 mg wet mass·mL^{-1}		
2	Rotenone (Sigma, R8875)	0.5 µM		Inhibition of Complex I
3	Succinate (Sigma, S2378)	10 mM	LEAK (*L*)	Substrate of Complex II
4	ADP Mg^{2+} (Merck, 117105)	5 mM	OXPHOS (*P*)	Substrate of ATP synthase
5	Cytochrome *c* (Sigma, C7752)	10 µM	OXPHOS (P_c)	Evaluate outer mt-membrane integrity
6	CCCP (Sigma, C2759)	0.5 µM per step	ET (*E*)	Uncoupling of mitochondrial respiration
7	Antimycin A (Sigma, A8674)	2.5 µM		Inhibition of Complex III

Table S 3: Reasons for discard of the non-transplanted livers

Liver #	Reason for discard
1	Inadequate lactate clearance, high perfusate transaminases
2	Inadequate lactate clearance, high perfusate transaminases
3	Inadequate lactate clearance, high perfusate IL-6 levels
4	High perfusate transaminases
5	Inadequate lactate clearance
6	Technical problems related to NMP
7	Malignant tumor of the donor
8	Histology: fibrosis and steatosis; prolonged cold ischemia time
9	Histology: fibrosis
10	High DRI; Histology: fibrosis; high perfusate transaminases
11	High risk organ (DCD); high perfusate transaminases
12	Histology: macrosteatosis, physiological perfusate pH could not be maintained despite $NaHCO_3$ addition, inadequate lactate clearance
13	Histology: fibrosis and steatosis
14	Histology: fibrosis and steatosis
15	Arteriosclerosis A. hepatica, inadequate lactate clearance

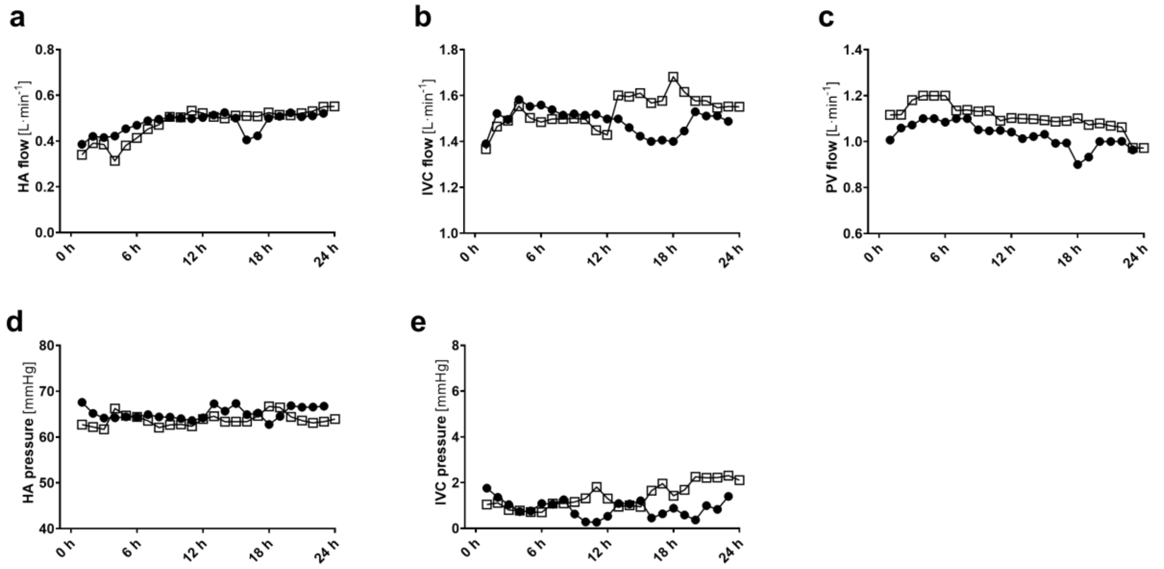

Figure S 1: Machine perfusion parameters during NMP. (a) Hepatic artery flow, (b) inferior vena cava flow, (c) portal vein flow, (d) hepatic artery pressure and (e) inferior vena cava pressure for livers transplanted (solid circles) and not transplanted (open boxes), expressed as median.

Table S 4: Semiquantitative assessment of histological changes during normothermic machine perfusion

Transplanted	pre	1 h	6 – 9 h	9 – 15 h	15 – 24 h	post
**Necrosis [median (IQR)]**	0 (0 – 0)	0 (0 – 1)	0 (0 – 1)	0 (0 – 1)	0 (0 – 1)	0 (0 – 1)
**Steatosis [median (IQR)]**	0 (0 – 1)	0 (0 – 1)	0 (0 – 1)	0 (0 – 1)	0 (0 – 0.75)	0 (0 – 1)
**Fibrosis [median (IQR)]**	0 (0 – 0)	0 (0 – 0)	0 (0 – 0)	0 (0 – 0)	0 (0 – 0)	0 (0 – 0)
**Inflammation [median (IQR)]**	1 (1 – 1)	1 (1 – 1)	1 (1 – 1)	1 (1 – 1)	1 (1 – 1)	1 (1 – 1)
**Vascular changes [median (IQR)]**	1 (0 – 1)	1 (0.75 – 1)	1 (1 – 1)	1 (1 – 1)	1 (1 – 1)	1 (1 – 1)

Discarded	pre	1 h	6 – 9 h	9 – 15 h	15 – 24 h
**Necrosis [median (IQR)]**	0 (0 – 0)	0 (0 – 1)	1 (0 – 1)	1.5 (1 – 3)	1.5 (0.75 – 3)
**Steatosis [median (IQR)]**	0.5 (0 – 1)	1 (0 – 1)	0.5 (0 – 1)	0.5 (0 – 1)	0.5 (0 – 1))
**Fibrosis [median (IQR)]**	0 (0 – 0)	0 (0 – 0)	0 (0 – 0)	0 (0 – 0.75)	0 (0 – 0.25)
**Inflammation [median (IQR)]**	1 (0 – 1)	1 (0.5 – 1)	1 (0 – 1)	1 (0.25 – 1)	1 (0 – 1)
**Vascular changes [median (IQR)]**	1 (0 – 1)	1 (0.5 – 1)	1 (0.25 – 1)	1 (1 – 1)	1 (1 – 1)

Table S 5: Semiquantitative assessment of immunohistochemistry during normothermic machine perfusion

	Transplanted			Discarded		
	**pre**	**6 – 9 h**	**end**	**pre**	**6 – 9 h**	**end**
**CD3 [median (IQR)]**	37.50 (15.00 – 127.50)	37.50 (14.25 – 75.00)	37.50 (14.25 – 127.50)	150.00 (52.50 – 232.50)	150.00 (45.00 – 225.00)	150.00 (45.00 – 225.00)
**CD20 [median (IQR)]**	7.50 (3.00 – 19.50)	4.50 (2.25 – 22.50)	4.50 (2.25 – 19.50)	15.00 (7.50 – 105.00)	60.00 (3.00 – 120.00)	60.00 (1.50 – 120.00)
**CD68 [median (IQR)]**	15.00 (3.00 – 30.00)	15.00 (3.00 – 18.75)	15.00 (3.00 – 30.00)	30.00 (42.50 – 22.50)	30.00 (22.50 – 42.50)	30.00 (22.50 – 65.00)
**TLR4 [median (IQR)]**	8.00 (0.00 – 40.00)	18.00 (0.00 – 40.00)	10.50 (0.00 – 31.50)	3.00 (0.00 – 15.00)	3.00 (0.00 – 15.00)	3.00 (0.00 – 15.00)
**CCasp3 [median (IQR)]**	0.00 (0.00 – 11.25)	0.00 (0.00 – 7.50)	0.00 (0.00 – 22.50)	0.00 (0.00 – 32.00)	4.00 (0.00 – 12.50)	4.00 (2.00 – 10.00)

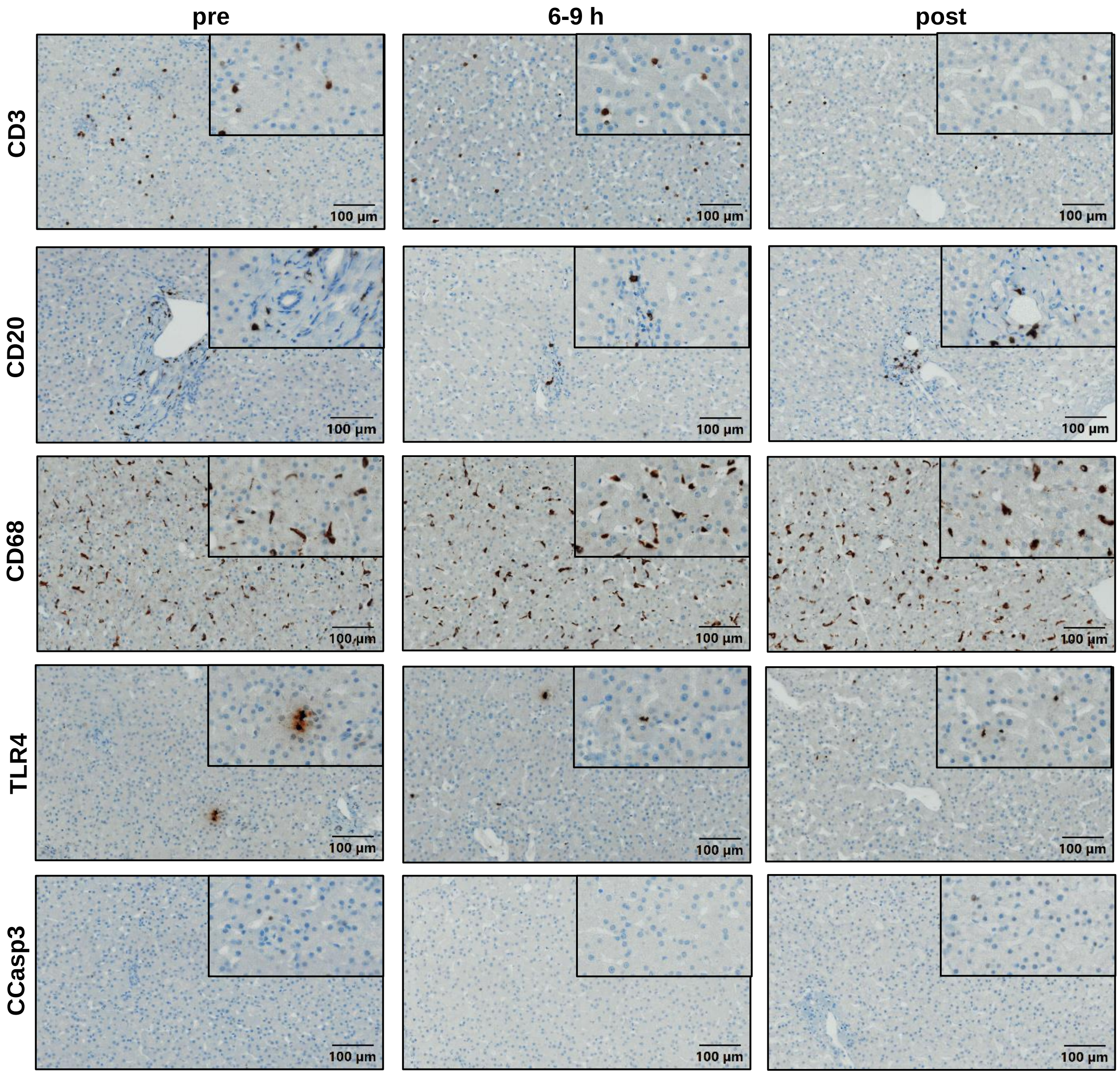

Figure S 2: Immunohistochemistry of livers with subsequent transplantation after NMP. Representative images of CD3, CD20, CD68, TLR4 and CCasp3 stainings in the course of NMP.

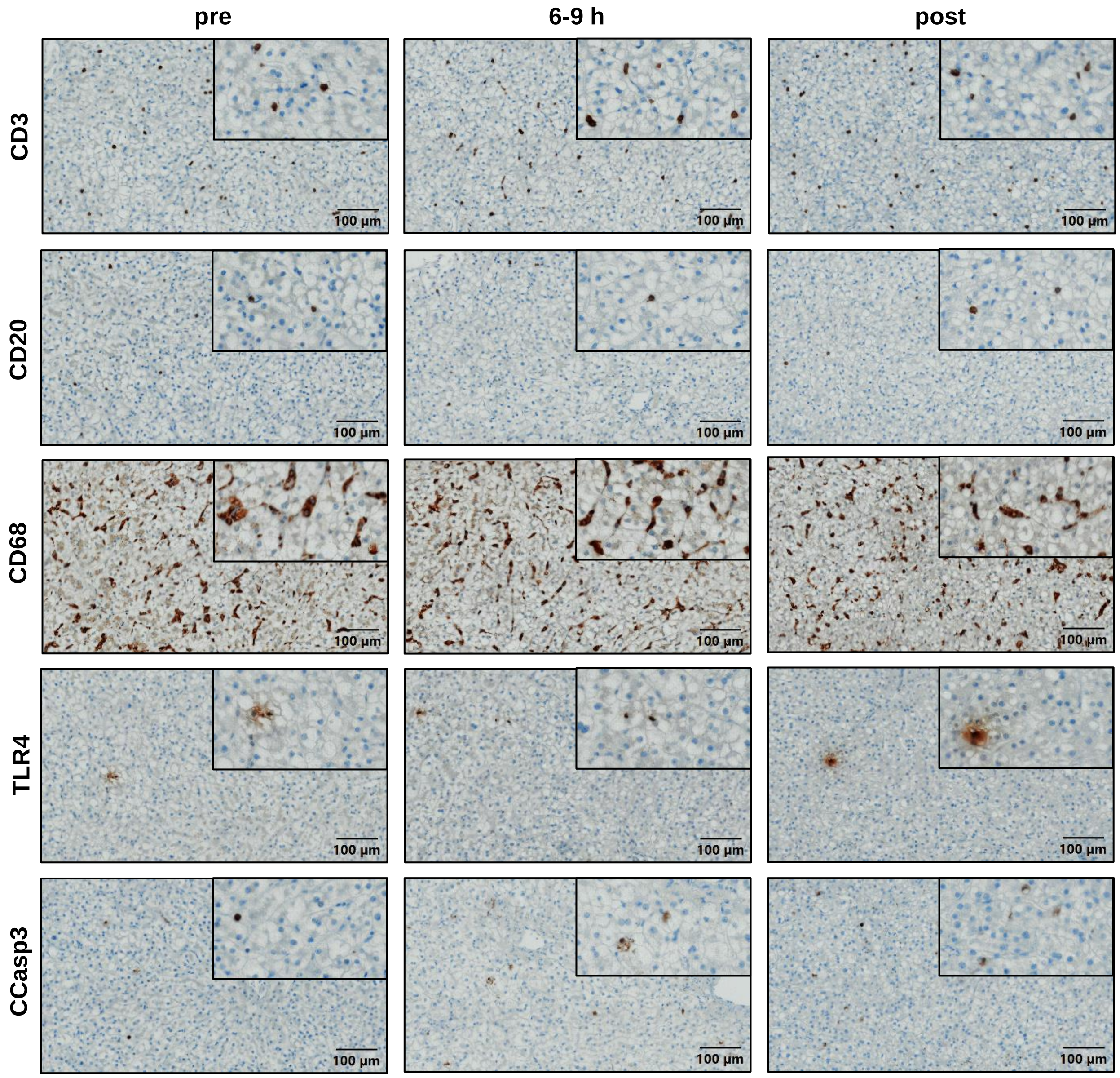

Figure S 3: Immunohistochemistry of livers discarded after NMP. Representative images of CD3, CD20, CD68, TLR4 and CCasp3 stainings in the course of NMP.

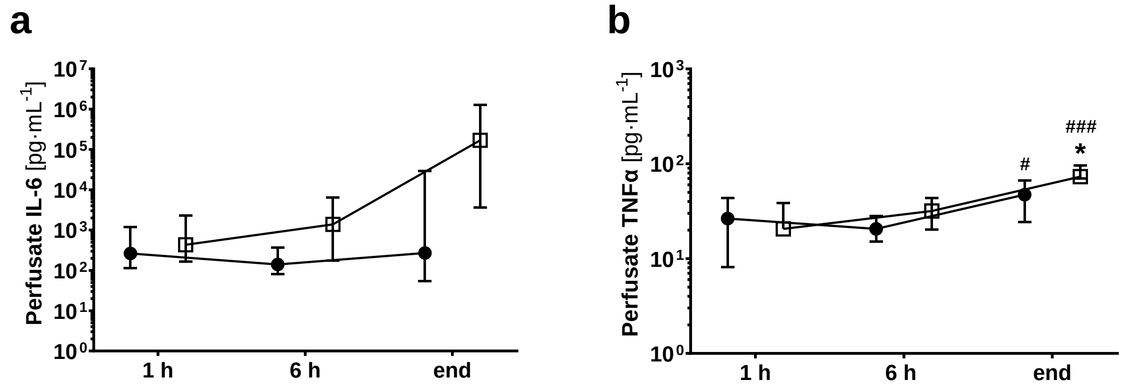

Figure S 4: Perfusate analysis during normothermic machine perfusion. (a) Interleukin-6 and (b) tumor necrosis factor alpha levels for transplanted (solid circles) and not transplanted (open boxes) livers, expressed as median and interquartile range. ($p < 0.05$, transplanted vs. not transplanted group; # $p < 0.05$, ### $p < 0.001$, compared to 1 h values within groups)*

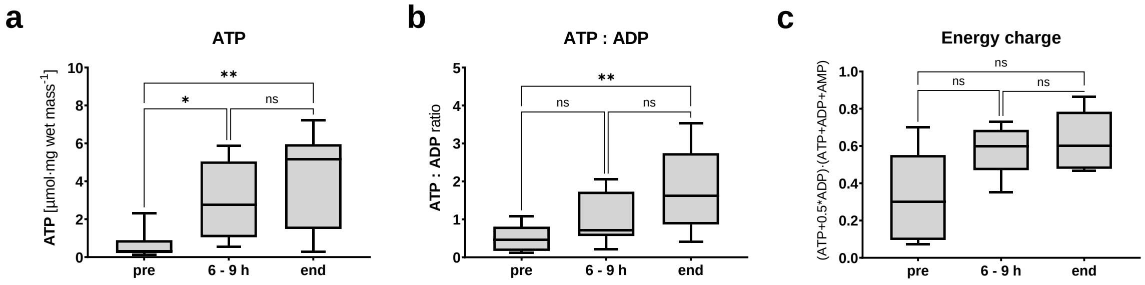

*Figure S 5: Analysis of adenine nucleotides in tissue biopsies during normothermic machine perfusion for not transplanted livers. (a) ATP concentration in liver biopsies; (b) Ratio of ATP to ADP; (c) Energy charge calculated as (ATP + 0.5*ADP)/(ATP + ADP + AMP). Results are shown as median, interquartile range, and min-max values. (* $p < 0.05$, ** $p < 0.01$, ns = no significance; comparisons between time points)*

Table S 6: ANOVA Table (Type II tests)

**Parameter**	**Sum Sq**	**Df**	***F***	***p*-value**
pre_P	0.85	1	3.39	0.317
h1_P	3.62	1	14.38	0.164
h6_P	0.07	1	0.28	0.692
pre_PL	0.64	1	2.56	0.356
pre_Jc	1.01	1	4.01	0.295
h1_Jc	1.77	1	7.06	0.229
h6_Jc	0.06	1	0.24	0.709
pre_L	0.89	1	3.53	0.311
h1_L	0.18	1	0.70	0.557
h6_L	0.32	1	1.26	0.463
pre_EP	0.19	1	0.77	0.541
h1_EP	0.18	1	0.72	0.551
h6_EP	0.35	1	1.40	0.447
pre_Pc	1.05	1	4.18	0.290
h1_Pc	0.81	1	3.20	0.324
h6_Pc	0.03	1	0.11	0.795
pre_E	1.56	1	6.20	0.243
h1_E	1.62	1	6.44	0.239
h6_E	0.20	1	0.81	0.533
Residuals	0.25	1		

Table S 7: ANOVA Table (Type II tests) - MEAF

**Parameter**	***b***	**conf**	***beta***	***p*-value**
(Intercept)	-4.98	[9.3, -19.3]		0.470
PL.auc	1.38	[3.77, -1.02]	0.54	0.241
Jc.auc	3.17	[13.6, -7.31]	0.70	0.531
L.auc	0.0429	[0.176, -0.0906]	0.45	0.505
Jc.auc:L.auc	-0.001	[0.235, -0.237]	-0.01	0.993

r^2 = .32, ad.r^2 = 0.150, $F_{(4, 16)}$= 1.88, p = 0.164

Table S 8: ANOVA Table (Type II tests) - EAD

**Parameter**	***b***	**conf**	***beta***	***p*-value**
(Intercept)	9.85	[36.9, -13.5]		0.422
PL.auc	-2.09	[1.66, -6.51]	0.12	0.290
Jc.auc	3.1	[21.5, -12.8]	>20	0.706
L.auc	-0.0344	[0.21, -0.263]	0.97	0.762
Jc.auc:L.auc	-0.0338	[0.325, -0.423]	0.97	0.851

r^2 = .21, ad.r^2 = 0.02, $F_{(4, 16)}$= 1.09, p = 0.392
